# Discovery of novel PDE9A inhibitors with antioxidant activities for treatment of Alzheimer’s disease

**DOI:** 10.1080/14756366.2017.1412315

**Published:** 2017-12-22

**Authors:** Chen Zhang, Qian Zhou, Xu-Nian Wu, Ya-Dan Huang, Jie Zhou, Zengwei Lai, Yinuo Wu, Hai-Bin Luo

**Affiliations:** a School of Pharmaceutical Sciences, Sun Yat-Sen University, Guangzhou, PR China;; b State Key Laboratory for Chemistry and Molecular Engineering of Medicinal Resources, Guangxi Normal University, Guilin, PR China;; c Collaborative Innovation Center of High Performance Computing, National University of Defence Technology, Changsha, PR China

**Keywords:** Phosphodiesterase-9, multifunctional anti-AD agents, antioxidant activity, Alzheimer’s disease, molecular docking

## Abstract

Phosphodiesterase-9 (PDE9) is a promising target for treatment of Alzheimer’s disease (AD). To discover multifunctional anti-AD agents with capability of PDE9 inhibition and antioxidant activity, a series of novel pyrazolopyrimidinone derivatives, coupling with the pharmacophore of antioxidants such as ferulic and lipolic acids have been designed with the assistance of molecular docking and dynamics simulations. Twelve out of 14 synthesised compounds inhibited PDE9A with IC_50_ below 200 nM, and showed good antioxidant capacities in the ORAC assay. Compound **1h,** the most promising multifunctional anti-AD agent, had IC_50_ of 56 nM against PDE9A and good antioxidant ability (ORAC (trolox) = 3.3). The selectivity of **1h** over other PDEs was acceptable. In addition, **1h** showed no cytotoxicity to human neuroblastoma SH-SY5Y cells. The analysis on structure-activity relationship (SAR) and binding modes of the compounds may provide insight into further modification.

## Introduction

Alzheimer’s disease (AD) is the most common type of dementia, which is characterised by progressive memory loss, decline in language skills, and other neurodegenerative disorders^1^. According to Alzheimer’s Association (USA), about 46.8 million people worldwide are suffering from AD. The morbidity and mortality are high, especially for elder persons above 60 years old. The global societal economic cost for AD patients was estimated at $818 billion in 2015 and will rise to $1000 billion in 2018, and $2000 billion in 2030^2^. Currently, AD has been one of the most challenges for public health around the world, severely impacting lives of patients and their families.

The pathogenic mechanism of Alzheimer’s disease is complicated and still has not fully understood yet. Several factors, such as low level of acetylcholine, deposition of β-amyloid (Aβ), aggregation of Tau-protein, and oxidative stress have been regarded as important in the pathophysiology of AD^3^. Current drugs for the treatment of AD include three acetylcholinesterase (AChE) inhibitors (donepezil, rivastigmine, and galantamine) and one *N*-methyl-*d*-aspartate receptor antagonist (memantine)^4^. However, administration of these drugs only can improve cognition and dementia degree of AD patients, but not reverse the underlying progression. In recent decades, discovery of anti-AD candidate drugs mainly focus on the anti-Aβ pathology. However, most clinical trials using these candidates failed^5^. One important reason for this failure might be that the single targeted drugs were not able to control the complicated pathogenic progression of AD. Thus, development of multifunctional anti-AD agents which simultaneously hit more than single target in the AD pathophysiology, has become an important research area in recent years^6^^,^^7^, especially those hitting at non-Aβ targets.

Phosphodiesterases (PDEs) are a super-enzyme family in charge of hydrolysing the second messengers cyclic adenosine monophosphate (cAMP) and cyclic guanosine monophosphate (cGMP)^8^^,^^9^. According to their structural and functional properties, PDEs can be divided into 11 subfamilies (PDE1–PDE11). In brain, the levels of cAMP and cGMP are increased *via* the inhibition of PDEs, activating AC/cAMP/PKA, or NO/cGMP/PKG signalling pathway, and thus increasing the level of the cAMP response element-binding protein (CREB), enhancing synaptic transmission, and reducing cognitive deficit^10^. PDEs inhibitors of PDE1, PDE2, PDE4, PDE5, and PDE9 have been demonstrated to be effective in restoring cognitive deficits in some preclinical models and clinical trials of AD^11^.

Among all the PDE subfamilies, Phosphodiesterase 9 (PDE9) has unique advantages on AD therapy for its highest affinity with cGMP among all the PDE families and its high expression level in the cortex, hippocampus, basal ganglia, and cerebellum of brain^12^. Currently, the effects of PDE9 inhibitors in the AD animal models have been deeply explored. In the social and object recognition tasks of rodents, PDE9 inhibitor **BAY73–6691** enhanced the ability of acquisition, consolidation, and retention of long-term potentiation (LTP), which also improved scopolamine-induced passive avoidance deficit and MK-801-induced short-term memory deficits^13^^,^^14^. In comparison with AChE inhibitor donepezil that only enhanced short memory in the early LTP, **BAY73–6691** not only increased both early and late LTP in rat hippocampal slices, but also transformed the early LTP into late LTP^15^. In addition, **BAY73–6691** protected Aβ_25–35_-induced oxidative damage in hippocampus *via* the activation of the cGMP-related NO-dependent signalling^16^. Besides **BAY73–6691,** PDE9 inhibitors **PF-04447943** also exhibited memory improvement in several AD models^17^^,^^18^. Currently, **PF-04447943** has already completed six phase I clinical trials and one phase II trial of AD^19^. Despite good effects on memory improvement obtained by PDE9 inhibitors in the preclinical and clinical trials, the number of PDE9 inhibitors for treatment of AD is very limited^20^^,^^21^, especially those combining the PDE9 inhibition as well as hitting at other pathophysiology of AD simultaneously.

Oxidative stress is an important risk factor in the AD onset and progression^22^. The abnormality of redox state may participate in the neurodegenerative process, resulting in reactive oxygen species (ROS) mediated and reactive nitrogen species (RNS) mediated impairment in AD brain. Furthermore, the levels of oxidative markers of biomolecules including proteins, lipids, carbohydrates, and nucleic acids, as well as the antioxidant enzymes, were observed to be changed. Numbers of antioxidants such as resveratrol, ferulic acid have been proved to be neuroprotectants. However, results from the clinical trials suggested that only antioxidant efficacy was not sufficient to modify AD progression^23^. Antioxidants combining with additional pharmacological effects might combat the complicated pathogenic mechanism of AD more efficiently. Thus, numerous multifunctional anti-AD agents with antioxidant activity and also hitting other targets in the AD pathophysiology were developed. Some of them even went into clinical trials^24^. Vinpocetine, a moderate PDE1 inhibitor with antioxidant activity, significantly improved learning and memory in the streptozotocin infused AD rat models. Further studies demonstrated that both the regulation of cyclic nucleotide signalling and antioxidant mechanism were responsible for the memory improvement obtained by vinpocetine^25^. For unique advantages of PDE9 inhibition over other PDEs, development of PDE9 inhibitors with antioxidant activity, to regulate both cGMP signalling and antioxidant mechanism is of great importance^20^.

With our continuing interest in discovery of PDE9 inhibitors^26^, we reported here the design, synthesis, and biological evaluation of novel pyrazolopyrimidinone derivatives with both PDE9 inhibition and antioxidant activity. Molecular docking and dynamics simulation were applied at the beginning to choose potential lead compounds with appropriate binding modes and binding free energies, in order to reduce the next synthetic work and improve the efficiency of lead discovery.

## Materials and methods

### Chemistry

^1^H NMR and ^13^C NMR spectra were recorded on a Bruker BioSpin GmbH spectrometer (Bruker, Billerica, MA) at 400.1 and 100.6 MHz, respectively. Coupling constants (*J*) were reported in Hertz (Hz). Spectra were referenced internally to the residual proton resonance in CDCl_3_ (δ 7.26 ppm) or DMSO (δ 2.50 ppm) as the internal standard. High-resolution mass spectra (HRMS) were obtained on an IT-TOF mass spectrometer. Flash column chromatography was performed using silica gel (200–300 mesh). Thin-layer chromatography was performed on precoated silica gel F-254 plates (0.25 mm) and was visualised with UV light. Unless otherwise noted, all the starting materials and reagents were purchased from commercial suppliers and used directly without further purification.

### Cyclopentyl-4-cyano-1h-pyrazol-5-amine (M-3)

To a solution of cyclopentylhydrazinedihydrochloride **M-2** (2.7 g, 20 mmol) in ethanol (20 ml), triethylamine (7.1 g, 70 mmol) was added slowly at 0 °C. After stirring for 2 h, 2-(methoxymethylene)malononitrile **M-1** (2.4 g, 22 mmol) was added to the mixture dropwise. The mixture was then stirred at room temperature for 3 h and refluxed at 80 °C for 3 h. After the reaction was finished, ethanol was evaporated to half of its volume. Water was added and the resulted precipitate was collected as a brown solid. The brown solid was purified by recrystallisation with ethanol as the solvent, providing compound **M-3**^20^^,^^27^ as a yellow solid (3.0 g, 85%). ^1^H NMR (400 MHz, DMSO) δ 8.11 (s, 1H), 5.51 (d, *J* = 6.8 Hz, 1H), 4.50–4.38 (m, 1H), 3.39 (s, 1H), 2.03–1.93 (m, 2H), 1.88–1.79 (m, 2H), 1.76–1.68 (m, 2H), 1.63–1.53 (m, 2H). ^13^C NMR (101 MHz, DMSO) δ 157.33, 134.01, 114.78, 76.01, 62.18, 31.82, 23.64. HRMS (ESI-TOF) *m/z* [M + H]^+^ calcd for C_9_H_13_N_4_ 117.1143, found 117.1140.

### 5-Amino-1-cyclopentyl-1H-pyrazole-4-carboxamide (M-4)

To a solution of compound **M-3** (1.8 g, 10 mmol) in ethanol (15 ml), 30% hydrogen peroxide (1.5 ml), and 25% ammonium hydroxide (4.0 ml) were added sequentially. The mixture was stirred at room temperature for 1 h. When the reaction was finished, saturated sodium thiosulfate was added and a precipitate was formed. The precipitate was filtered, washed with water three times, and dried over vacuum to give compound **M-4**^20^^,^^27^ (1.7 g, 86%) as a white solide. ^1^H NMR (400 MHz, DMSO) δ 7.63 (s, 1H), 7.16 (br s, 1H), 6.62 (br s, 1H), 6.13 (m, 1H), 4.57–4.45 (m, 1H), 3.39 (s, 1H), 2.00–1.87 (m, 2H), 1.87–1.72 (m, 4H), 1.63–1.50 (m, 2H). ^13^C NMR (101 MHz, DMSO) δ 165.73, 148.24, 136.27, 96.17, 55.30, 30.65, 23.48. HRMS (ESI-TOF) *m/z* [M + H]^+^ calcd for C_9_H_15_N_4_O 195.1242, found 195.1246.

### (R)-benzyl(1–(1-cyclopentyl-4-oxo-4,5-dihydro-1H-pyrazolo[3,4-d]pyrimidin-6-yl)ethyl)carbamate (M-5)

NaH (60% dispersion in mineral oil, 0.80 g, 30 mmol) was added to the solution of **M-4** (1.9 g, 10 mmol) and (*R*)-ethyl-2-(((benzyloxy)carbonyl)amino)propanoate (7.5 g, 30 mmol) in anhydrous THF (40 ml). The mixture was stirred at room temperature overnight. Water was added slowly to the mixture. The mixture was then extracted with ethyl acetate three times, washed with brine, dried over Na_2_SO_4_, and concentrated. The residue was purified by silica gel chromatography (methanol/dichloromethane 1:100) to afford compound **M-5**^20^ as a yellow oil (2.0 g, 42%). ^1^H NMR (400 MHz,CDCl_3_) δ 11.90 (s, 1H), 8.05 (s, 1H), 7.32–7.28 (m, 5H), 5.98 (d, *J* = 6.1 Hz, 1H), 5.23–5.05 (m,3H), 4.98–4.83 (m, 1H), 2.10 (dd, *J* = 13.6, 6.9 Hz, 4H), 2.02–1.92 (m, 2H), 1.79–1.69 (m, 2H), 1.60 (d, *J* = 7.1 Hz, 3H). ^13^C NMR (101 MHz, CDCl_3_) δ 160.26, 159.79, 155.83, 151.90, 136.12, 134.56, 128.50, 128.18, 104.41, 67.18, 57.89, 50.43, 32.47, 24.78, 20.56. HRMS (ESI-TOF) *m/z* [M + H]^+^ calcd for C_20_H_24_N_5_O_3_ 382.1880, found 382.1879.

### (R)-6–(1-aminoethyl)-1-cyclopentyl-1H-pyrazolo[3,4-d]pyrimidin-4(5H)-one (M-6)

To a solution of compound **M-5** (3.8 g, 10 mmol) in methanol, Pd/C (10%, 0.4 g) was added. The mixture was stirred at room temperature for 24 h under an atmospheric pressure of hydrogen. The catalyst was filtered and the filtrate was concentrated. The residue was purified by silica gel chromatography (methanol/dichloromethane 1:50) to afford compound **M-6**^20^ as a yellow solid (1.3 g, 52%). ^1^H NMR (400 MHz, CDCl_3_) *δ* 8.05 (s, 1H), 5.22–5.08 (m, 1H), 4.12 (q, *J* = 6.8 Hz, 1H), 2.20–2.03 (m, 4H), 2.01–1.91 (m, 2H), 1.76–1.67 (m, 2H), 1.53 (d, *J* = 6.9 Hz, 3H). ^13^C NMR (101 MHz, CDCl_3_) *δ* 162.65, 158.53, 152.48, 134.49, 104.72, 57.81, 49.70, 32.44, 32.32, 24.75, 23.15. HRMS (ESI-TOF) *m/z* [M + H]^+^ calcd for C_12_H_17_N_5_O 247.1438, found 247.1433.

### General procedures for the preparation of compound 1a-1l, 2, and 3

A mixture of compound **M-6** (0.25 g, 1.0 mmol), EDC.HCl (0.15 g, 1.5 mmol), DMAP (0.002 g, 0.02 mmol), and the corresponding acid (1.0 mmol) was stirred at room temperature overnight^28^. Water was added to the mixture. The mixture was then extracted with ethyl acetate three times, washed with brine, dried over Na_2_SO_4_, and concentrated. The resulting residue was purified by silica gel chromatography and eluted with methanol and dichloromethane (1:100) to afford the final product.

### (R, E)-N-(1–(1-cyclopentyl-4-oxo-4, 5-dihydro-1H-pyrazolo[3,4-d]pyrimidin-6-yl)ethyl)-3–(4-hydroxy-3-methoxyphenyl)acrylamide (1a)

Compound **1a** (0.18 g, 42%) was synthesised from ferulic acid as a white solid. ^1^H NMR (400 MHz, CDCl_3_) *δ* 11.98 (s, 1H), 8.10 (s, 1H), 7.59 (dd, *J* = 15.6, 2.9 Hz, 1H), 6.99 (d, *J* = 8.2 Hz, 1H), 6.94 (t, *J* = 5.3 Hz, 2H), 6.85 (d, *J* = 8.2 Hz, 1H), 6.32 (t, *J* = 14.4 Hz, 1H), 5.99 (d, *J* = 63.7 Hz, 1H), 5.35–5.26 (m, 1H), 5.26–5.17 (m, 1H), 3.84 (s, 3H), 2.22–2.11 (m, 2H), 2.10–2.01 (m, 2H), 2.00–1.90 (m, 2H), 1.79–1.69 (m, 2H), 1.66 (d, *J* = 7.1 Hz, 3H). ^13^C NMR (101 MHz, DMSO) *δ* 165.89, 162.06, 158.37, 152.03, 148.93, 148.29, 140.26, 134.46, 126.71, 122.16, 118.75, 116.15, 111.27, 104.76, 57.62, 56.20, 55.97, 48.39, 32.66, 32.44, 24.76, 19.78. HRMS (ESI-TOF) *m/z* [M + H]^+^ calcd for C_22_H_25_N_5_O_4_ 424.1979, found 424.1985.

### (R, E)-N-(1–(1-cyclopentyl-4-oxo-4,5-dihydro-1H-pyrazolo[3,4-d]pyrimidin-6-yl)ethyl)-3–(3,4-dihydroxyphenyl)acrylamide (1b)

Compound **1b** (0.19 g, 45%) was synthesised from caffeic acid as a white solid. ^1^H NMR (400 MHz, DMSO) *δ* 9.31 (s, 2H), 8.52 (d, *J* = 6.6 Hz, 1H), 8.07 (s, 1H), 7.30 (d, *J* = 15.7 Hz, 1H), 6.90 (d, *J* = 8.2 Hz, 1H), 6.80 (d, *J* = 8.1 Hz, 1H), 6.52 (d, *J* = 15.7 Hz, 1H), 5.20–5.08 (m, 1H), 4.91 (p, *J* = 6.7 Hz, 1H), 2.09 (d, *J* = 11.9 Hz, 2H), 2.03–1.94 (m, 2H), 1.92 (d, *J* = 16.4 Hz, 2H), 1.69 (s, 2H), 1.48 (t, *J* = 8.9 Hz, 3H). ^13^C NMR (101 MHz, DMSO) *δ* 165.89, 162.06, 158.37, 152.03, 148.93, 148.29, 140.26, 134.46, 126.71, 122.16, 118.75, 116.15, 111.27, 104.76, 57.62, 56.20, 55.97, 48.39, 32.66, 32.44, 24.76, 19.78. HRMS (ESI-TOF) *m/z* [M + H]^+^ calcd for C_21_H_23_N_5_O_4_ 410.1823, found 410.1827.

### (R, E)-N-(1–(1-cyclopentyl-4-oxo-4, 5-dihydro-1H-pyrazolo[3,4-d]pyrimidin-6-yl) ethyl)-3– (3,4-dimethoxyphenyl)acrylamide (1c)

Compound **1c** (0.26 g, 59%) was synthesised from 3,4-dimethoxycinnamic acid as a white solid. ^1^H NMR (400 MHz, CDCl_3_) *δ* 12.12 (s, 1H), 8.09 (s, 1H), 7.62 (dd, *J* = 15.6, 2.8 Hz, 1H), 7.11 (d, *J* = 7.8 Hz, 1H), 7.03 (d, *J* = 8.3 Hz, 1H), 6.96 (s, 1H), 6.79 (d, *J* = 8.4 Hz, 1H), 6.39 (d, *J* = 15.6 Hz, 1H), 5.31 (dd, *J* = 14.5, 7.2 Hz, 1H), 5.22 (m, 1H), 3.88 (s, 3H), 3.83 (s, 3H), 2.10 (m, 4H), 1.98 (m, 2H), 1.73 (m, 2H), 1.66 (d, *J* = 7.0 Hz, 3H). ^13^C NMR (101 MHz, CDCl_3_) *δ* 166.34, 160.21, 159.99, 151.99, 150.79, 149.08, 142.28, 134.25, 127.44, 122.21, 117.46, 111.02, 109.71, 104.46, 57.90, 55.92, 55.85, 49.03, 32.49, 24.80, 20.10. HRMS (ESI-TOF) *m/z* [M + H]^+^ calcd for C_23_H_27_N_5_O_4_ 438.2136, found 438.2143.

### (R, E)-N-(1–(1-cyclopentyl-4-oxo-4, 5-dihydro-1H-pyrazolo[3,4-d]pyrimidin-6-yl)ethyl)-3–(3-hydroxy-4- methoxyphenyl)acrylamide (1d)

Compound **1d** (0.19 g, 45%) was synthesised from 3-hydroxy-4-methoxycinnamic acid as a white solid. ^1^H NMR (400 MHz, CDCl_3_) *δ* 11.81 (s, 1H), 8.09 (s, 1H), 7.60 (d, *J* = 15.4 Hz, 1H), 7.00 (d, *J* = 6.7 Hz, 1H), 6.94 (s, 1H), 6.87 (m, 1H), 6.33 (d, *J* = 15.1 Hz, 1H), 5.99 (s, 1H), 5.30 (dd, *J* = 15.1, 8.8 Hz, 1H), 5.20 (dd, *J* = 16.8, 10.1 Hz, 1H), 3.85 (s, 3H), 2.15 (m, 4H), 1.98 (m, 2H), 1.78 (m, 2H), 1.66 (d, *J* = 4.5 Hz, 3H). ^13^C NMR (101 MHz, Acetone) *δ* 166.53, 160.67, 157.50, 151.74, 148.62, 147.75, 141.14, 133.78, 127.00, 121.97, 117.79, 115.28, 110.54, 104.79, 57.67, 55.33, 48.40, 32.11, 24.51, 17.85. HRMS (ESI-TOF) *m/z* [M + H]^+^ calcd for C_22_H_25_N_5_O_4_ 424.1979, found 424.1986.

### (R, E)-N-(1–(1-cyclopentyl-4-oxo-4, 5-dihydro-1H-pyrazolo[3,4-d]pyrimidin-6-yl)ethyl) -3–(3-hydroxy-2,4-dimethoxyphenyl)acrylamide (1e)

Compound **1e** (0.23 g, 51%) was synthesised from 3-hydroxy-2, 4-dimethoxycinnamic acid as a white solid. ^1^H NMR (400 MHz, CDCl_3_) *δ* 11.66 (s, 1H), 8.08 (s, 1H), 7.58 (d, *J* = 15.4 Hz, 1H), 6.77 (d, *J* = 7.0 Hz, 1H), 6.72 (s, 1H), 6.35 (d, *J* = 15.4 Hz, 1H), 5.78 (s, 1H), 5.24 (m, 2H), 3.87 (s, 6H), 2.11 (m, 4H), 1.99 (m, 2H), 1.73 (d, *J* = 4.3 Hz, 2H), 1.67 (d, *J* = 6.1 Hz, 4H). ^13^C NMR (101 MHz, CDCl_3_) *δ* 166.41, 159.82, 159.68, 151.92, 147.18, 142.90, 136.89, 136.49, 134.27, 125.95, 117.31, 115.48, 104.93, 104.58, 57.96, 56.33, 48.91, 32.51, 24.82, 19.60. HRMS (ESI-TOF) *m/z* [M + H]^+^ calcd for C_23_H_27_N_5_O_5_ 454.2085, found 454.2082.

### (R, E)-N-(1–(1-cyclopentyl-4-oxo-4, 5-dihydro-1H-pyrazolo[3,4-d]pyrimidin-6-yl) ethyl)-3–(3-methoxyphenyl)acrylamide (1f)

Compound **1f** (0.19 g, 47%) was synthesised from 3-methoxycinnamic acid as a white solid. ^1^H NMR (400 MHz, DMSO) *δ* 8.60 (d, *J* = 5.4 Hz, 1H), 8.03 (s, 1H), 7.49–7.28 (m, 2H), 7.22–7.06 (m, 2H), 6.97 (d, *J* = 7.2 Hz, 1H), 6.78 (d, *J* = 15.7 Hz, 1H), 5.21–4.99 (m, 1H), 4.95–4.77 (m, 1H), 3.79 (s, 3H), 2.04 (s, 2H), 1.90 (d, *J* = 34.6 Hz, 4H), 1.64 (s, 2H), 1.46 (d, *J* = 6.0 Hz, 3H). ^13^C NMR (101 MHz, DMSO) *δ* 165.41, 161.86, 160.06, 158.39, 152.09, 139.76, 136.67, 134.44, 130.49, 122.41, 120.43, 115.88, 113.13, 104.78, 57.67, 55.59, 48.42, 32.64, 32.43, 24.76. HRMS (ESI-TOF) *m/z* [M + H]^+^ calcd for C_22_H_25_N_5_O_3_ 408.2030, found 408.2033.

### (R, E)-N-(1–(1-cyclopentyl-4-oxo-4, 5-dihydro-1H-pyrazolo[3,4-d]pyrimidin-6-yl)ethyl)-3–(4-hydroxyphenyl)acrylamide (1g)

Compound **1g** (0.21 g, 52%) was synthesised from 4-hydroxycinnamic acid as a white solid. ^1^H NMR (400 MHz, DMSO) *δ* 12.08 (s, 1H), 9.90 (s, 1H), 8.46 (d, *J* = 5.9 Hz, 1H), 8.02 (s, 1H), 7.50–7.35 (m, 2H), 7.34 (d, *J* = 15.7 Hz, 1H), 6.80 (d, *J* = 7.7 Hz, 2H), 6.55 (d, *J* = 15.9 Hz, 1H), 5.23–4.96 (m, 1H), 4.94–4.68 (m, 1H), 2.03 (s, 2H), 1.91 (d, *J* = 37.4 Hz, 4H), 1.64 (s, 2H), 1.45 (d, *J* = 6.4 Hz, 3H). ^13^C NMR (101 MHz, DMSO) *δ* 165.92, 162.08, 159.48, 158.39, 152.02, 140.00, 134.44, 129.83, 126.20, 118.38, 116.25, 104.75, 60.24, 57.60, 48.39, 32.65, 32.43, 24.76, 19.75. HRMS (ESI-TOF) *m/z* [M + H]^+^ calcd for C_21_H_23_N_5_O_3_ 394.1874, found 394.1866.

### (R, E)-N-(1–(1-cyclopentyl-4-oxo-4, 5-dihydro-1H-pyrazolo[3,4-d]pyrimidin-6-yl)ethyl)-3–(3-hydroxyphenyl)acrylamide (1h)

Compound **1h** (0.15 g, 39%) was synthesised from 3-hydroxycinnamic acid as a white solid. ^1^H NMR (400 MHz, DMSO) *δ* 12.08 (s, 1H), 9.90 (s, 1H), 8.46 (d, *J* = 5.9 Hz, 1H), 8.02 (s, 1H), 7.50–7.35 (m, 2H), 7.34 (d, *J* = 15.7 Hz, 1H), 6.80 (d, *J* = 7.7 Hz, 2H), 6.55 (d, *J* = 15.9 Hz, 1H), 5.23–4.96 (m, 1H), 4.94–4.68 (m, 1H), 2.03 (s, 2H), 1.91 (d, *J* = 37.4 Hz, 4H), 1.64 (s, 2H), 1.45 (d, *J* = 6.4 Hz, 3H). ^13^C NMR (101 MHz, DMSO) *δ* 165.92, 162.08, 159.48, 158.39, 152.02, 140.00, 134.44, 129.83, 126.20, 118.38, 116.25, 104.75, 60.24, 57.60, 48.39, 32.65, 32.43, 24.76, 19.75. HRMS (ESI-TOF) *m/z* [M + H]^+^ calcd for C_21_H_23_N_5_O_3_ 394.1874, found 394.1871.

### (R, E)-N-(1–(1-cyclopentyl-4-oxo-4, 5-dihydro-1H-pyrazolo[3,4-d]pyrimidin-6-yl)ethyl)-3–(4-(dimethylamino)phenyl)acrylamide (1i)

Compound **1i** (0.27 g, 63%) was synthesised from 4-hydroxycinnamic acid as a white solid. ^1^H NMR (400 MHz, DMSO) *δ* 12.10 (s, 1H), 9.62 (s, 1H), 8.60 (d, *J* = 6.7 Hz, 1H), 8.02 (s, 1H), 7.34 (d, *J* = 15.8 Hz, 1H), 7.21 (d, *J* = 7.8 Hz, 1H), 7.00 (d, *J* = 7.7 Hz, 1H), 6.95 (s, 1H), 6.80 (dd, *J* = 8.0, 1.8 Hz, 1H), 6.70 (d, *J* = 15.8 Hz, 1H), 5.11 (m, 1H), 4.89 (m, 1H), 2.51 (d, *J* = 1.6 Hz, 2H), 2.05 (m, 2H), 1.94 (m, 2H), 1.86 (m, 2H), 1.64 (m, 2H), 1.46 (d, *J* = 7.0 Hz, 3H). ^13^C NMR (101 MHz, DMSO) *δ* 165.47, 161.95, 158.37, 158.18, 152.01, 140.01, 136.49, 134.44, 130.45, 121.82, 119.27, 117.32, 114.20, 104.77, 57.68, 48.43, 32.63, 32.42, 24.75, 19.72. HRMS (ESI-TOF) *m/z* [M + H]^+^ calcd for C_23_H_28_N_6_O_2_ 421.2347, found 421.2350.

### (R, E)-N-3–(3-chlorophenyl)-N-(1–(1-cyclopentyl-4-oxo-4,5-dihydro-1H-pyrazolo[3,4-d] pyrimidin-6-yl)ethyl)acrylamide (1j)

Compound **1j** (0.19 g, 45%) was synthesised from 3-chlorolcinnamic acid as a white solid. ^1^H NMR (400 MHz, DMSO) *δ* 8.65 (d, *J* = 6.6 Hz, 1H), 8.02 (s, 1H), 7.65 (s, 1H), 7.56 (d, *J* = 4.9 Hz, 1H), 7.43 (t, *J* = 11.7 Hz, 3H), 6.83 (d, *J* = 15.9 Hz, 1H), 5.15–5.02 (m, 1H), 4.94–4.80 (m, 1H), 2.04 (d, *J* = 12.2 Hz, 2H), 1.98–1.74 (m, 4H), 1.63 (s, 2H), 1.46 (d, *J* = 6.9 Hz, 3H). ^13^CNMR (101 MHz, DMSO) *δ* 165.17, 161.76, 158.45, 151.96, 138.34, 137.47, 134.45, 134.16, 131.30, 129.75, 127.70, 126.58, 123.64, 104.73, 57.71, 48.46, 32.61, 32.41, 24.75, 19.72. HRMS (ESI-TOF) *m/z* [M + H]^+^ calcd for C_21_H_22_ClN_5_O_2_ 412.1535, found 412.1540.

### (R, E)-N-(1–(1-cyclopentyl-4-oxo-4, 5-dihydro-1H-pyrazolo[3,4-d]pyrimidin-6-yl)ethyl)-3–(4-(trifluoromethyl)phenyl)acrylamide (1k)

Compound **1k** (0.17 g, 39%) was synthesised from 4-trifluoromethylcinnamic acid as a white solid. ^1^H NMR (400 MHz, DMSO) *δ* 12.13 (s, 1H), 8.71 (d, *J* = 6.8 Hz, 1H), 8.03 (s, 1H), 7.80 (s, 4H), 7.52 (d, *J* = 15.9 Hz, 1H), 6.92 (d, *J* = 15.9 Hz, 1H), 5.18–5.04 (m, 1H), 4.96–4.83 (m, 1H), 2.13–2.00 (m, 2H), 1.95 (dd, *J* = 17.8, 11.5 Hz, 2H), 1.86 (dd, *J* = 9.8, 6.9 Hz, 2H), 1.64 (s, 2H), 1.47 (d, *J* = 7.0 Hz, 3H). ^13^C NMR (400 MHz, DMSO) *δ* 166.67, 163.65, 159.42, 156.07, 149.85, 141.77, 135.38, 130.64, 126.91, 121.90, 116.23, 112.07, 61.59, 48.95, 36.17, 25.81, 22.00. HRMS (ESI-TOF) *m/z* [M + H]^+^ calcd for C_22_H_22_F_3_N_5_O_2_ 446.1798, found 446.1794.

### (R, E)-N-(1–(1-cyclopentyl-4-oxo-4, 5-dihydro-1H-pyrazolo[3,4-d]pyrimidin-6-yl)ethyl)-2–(3,4-dihydroxyphenyl)acetamide (1l)

Compound **1l** (0.22 g, 56%) was synthesised from 3, 4-dihydroxyphenylacetic acid as a white solid. ^1^H NMR (400 MHz, DMSO) *δ* 12.06 (s, 1H), 8.74 (d, *J* = 19.1 Hz, 2H), 8.34 (d, *J* = 6.2 Hz, 1H), 8.00 (s, 1H), 6.85–6.56 (m, 2H), 6.53 (d, *J* = 7.5 Hz, 1H), 5.09–4.84 (m, 1H), 4.84–4.53 (m, 1H), 3.33–3.24 (m, 2H), 2.03 (s, 2H), 1.88 (s, 4H), 1.68 (s, 2H), 1.39 (d, *J* = 6.7 Hz, 3H). ^13^C NMR (101 MHz, DMSO) *δ* 171.07, 161.94, 158.36, 151.92, 145.39, 144.33, 134.42, 127.12, 120.22, 117.10, 115.63, 104.63, 57.39, 48.00, 41.90, 32.81, 32.38, 24.84, 19.86. HRMS (ESI-TOF) *m/z* [M + H]^+^ calcd for C_20_H_23_N_5_O_4_ 398.1823, found 398.1826.

### N-((1N)-1–(1-cyclopentyl-4-oxo-3a, 4, 5, 6-tetrahydro-1H-pyrazolo[3,4-d]pyrimi–din-6-yl)ethyl)-5-((R)-1,2-dithiolan-3-yl)pentanamide (2)

Compound **2** (0.21 g, 48%) was synthesised from (*R*)-*α*-lipoic acid as a white solid. ^1^H NMR (400 MHz, DMSO) *δ* 12.03 (s, 1H), 8.32 (d, *J* = 6.7 Hz, 1H), 8.02 (s, 1H), 5.11 (s, 1H), 4.73 (s, 1H), 3.60 (dd, *J* = 8.4, 5.9 Hz, 1H), 3.14 (dd, *J* = 13.5, 7.2 Hz, 2H), 2.38 (s, 1H), 2.20 (s, 2H), 2.04 (s, 2H), 2.01–1.75 (m, 5H), 1.75–1.61 (m, 3H), 1.59–1.45 (m, 3H), 1.38 (d, *J* = 7.0 Hz, 5H). ^13^C NMR (101 MHz, DMSO) *δ* 172.71, 162.26, 158.36, 152.07, 134.44, 104.70, 57.54, 56.50, 48.06, 38.55, 35.32, 34.55, 32.55, 28.74, 25.38, 24.78, 19.57. HRMS (ESI-TOF) *m/z* [M + H]^+^ calcd for C_20_H_29_N_5_O_2_S_2_ 436.1835, found 436.1830.

### N-((R)-1–(1-cyclopentyl-4-oxo-4, 5-dihydro-1H-pyrazolo[3,4-d]pyrimidin-6-yl)ethyl) -6-hydroxy-2,5,7,8-tetramethylchromane-2-carboxamide (3)

Compound **3** (0.17 g, 35%) was synthesised from trolox as a white solid. ^1^H NMR (400 MHz, CDCl_3_) *δ* 8.02 (d, *J* = 17.0 Hz, 1H), 5.16 (m, 1H), 4.98 (m, 1H), 2.81 (s, 3H), 2.63 (dd, *J* = 16.2, 9.2 Hz, 2H), 2.35 (dd, *J* = 13.2, 6.7 Hz, 1H), 2.25 (s, 1H), 2.18 (m, 3H), 2.12 (s, 3H), 2.05 (d, *J* = 3.7 Hz, 3H), 1.98 (m, 2H), 1.73 (m, 2H), 1.61 (s, 6H), 1.59 (s, 1H). ^13^C NMR (101 MHz, CDCl_3_) *δ* 174.78, 159.43, 151.72, 145.88, 144.19, 134.71, 121.77, 119.52, 119.34, 117.86, 104.55, 99.99, 57.71, 47.94, 38.66, 32.57, 29.72, 24.83, 23.72, 20.43, 19.64, 12.39, 12.02, 11.45. HRMS (ESI-TOF) *m/z* [M + H]^+^ calcd for C_26_H_33_N_5_O_4_ 480.2605, found 480.2615.

### *In vitro* assay for PDE9 inhibitors

#### Protein expression and purification

The recombinant pET-PDE9A2 plasmid (catalytic domain, 181–506) was subcloned and purified in the same protocols we reported previously^26^ and then transferred into the *E. coli* strain BL21 (Codonplus, Stratagene, San Diego, CA). The *E. coli* cells carrying pET-PDE9A2 was grown in LB medium (containing 100 μg/ml ampicillin and 0.4% glucose) at 37 °C until OD_600_ = 0.6–0.8. 0.1 mM iso-propyl-β-D-thiogalactopyranoside was added to induce the PDE9A2 protein expression at 15 °C for 20 h. PDE9A was purified on the nickel nitriloacetic acid (Ni-NTA) column (Qiagen, Hilden, Germany) and eluted with 0.25 M imidazole. The concentration of PDE9A was estimated by the absorbance at 280 nm (calculated using ProtParam software, http://web.expasy.org/protparam). A typical batch of purification yielded 30–60 mg PDE9A2 protein from a 0.5 l cell culture. The PDE9A2 proteins had purity greater than 90% as shown by sodium dodecyl sulphate polyacrylamide gel electrophoresis (SDS-PAGE).

#### Enzymatic assays against PDE9A

The enzymatic activities of the catalytic domain of PDE9A2 were measured by using ^3^H-cGMP as the substrate and the assay buffer of 50 mM Tris-HCl (pH 8.0), 10 mM MgCl_2_, and 1 mM DTT. Each assay used ^3^H-cGMP 20,000–30,000 cpm. The reaction was carried out at room temperature (25 °C) for 15 min and then terminated by addition of 0.2 M ZnSO_4_. The reaction product was precipitated by 0.2 N Ba(OH)_2_, whereas unreacted ^3^H-cGMP remained in the supernatant. The radioactivity in the supernatant was measured in 2.5 ml of Ultima Gold liquid scintillation cocktails (PerkinElmer, Waltham, MA) using a PerkinElmer 2910 liquid scintillation counter. At least eight concentrations of inhibitors were measured parallel in each measurement, resulting an inhibition curve to calculate the IC_50_ value of inhibitors by nonlinear regression. Each measurement was repeated at least three times. The mean and standard deviation statistics of these three IC_50_ values were used as the final IC_50_ value and the corresponding SD. **BAY73–6691** purchased from SIGMA (Mendota Heights, MN) was used as the reference compound.

### *In vitro* assay for inhibition of the lead compounds on PDE1B and PDE8A

The catalytic domains of PDE1B (10–516) and PDE8A (480–820) were purified with the published protocol^29^^,^^30^. The enzymatic activities of PDE1B and PDE8A were assayed by using ^3^H-cGMP and ^3^H-cAMP as the substrates, respectively, following the same procedure for PDE9A.

### Antioxidant activity assay

The antioxidant activity was determined by the modified oxygen radical absorbance capacity fluorescein (ORAC-FL) method^31^^,^^32^. All the assays were diluted with 75 mM phosphate buffer (pH 7.4). The final reaction mixture was 200 μl. Test compound (20 μl) and fluorescein (120 μl, 150 nM final concentration) were placed in the wells of a black 96-well plate. After the mixture was incubated at 37 °C for 15 min, AAPH solution (60 μl, 12 mM final concentration) was added. The plate was placed in a Spectrafluor Plus plate reader (Tecan, Crailsheim, Germany) and the fluorescence was recorded every minute for 4 h with excitation at 485 nm and emission at 535 nm. Trolox was used as standard (1–8 μM, final concentration). A blank (FL + AAPH) in phosphate buffer instead of test compounds and trolox calibration were performed in each assay. The samples were measured at different concentration (1–10 μM). All the reaction mixture was prepared fourfold and at least three independent assays were performed for each sample. Fluorescence in time course was normalised on basis of the blank (without antioxidants). The ORAC-FL values were calculated as the reported method. ORAC-FL values were expressed as trolox equivalents.

### *In vitro* cell viability assay

SH-SY5Y cells were used for the cytotoxicity test by the MTT (3–(4, 5-dimethyl-2-thiazolyl)-2,5-diphenyl-2-H-tetrazoliumbromide) method^33^. After incubation of the SH-SY5Y cells (5 × 10^3^) in a 96-well plate for 24 h, compounds at concentrations of 10, 20, 40, 60, 80, and 100 μM, were added. After incubation for 48 h, MTT solution was added to each cell well and the system was further inoculated for 4 h at 37 °C. The purple formazan dye and 120 μl DMSO were added to the cells and the absorbance was measured at 570 nm using EnSpire-2300 Multimode Reader (PerkinElmer, Waltham, MA).

### Molecular docking

The crystal structure of PDE9A in the complex with a highly selective inhibitor **28** (PDB ID:4GH6) was used in this study^34^. Surflex-dock^35^ embedded in the software Tripos Sybyl version 2.0 (Tripos Software, Inc., El Cerrito, CA) was used for molecular docking. Two metal ions in this crystal structure were kept as they were crucial for the PDE’s catalytic activity. Most of the water molecules in this crystal structure were removed except the ones near these two metal ions. Hydrogen atoms were added and the ionisable residues were protonated at the neutral pH. The protomol was generated using a ligand-based approach and the bound ligand was used for protomol generation. The proto_thresh and proto_bloat parameters represent how much the protomol can be buried in the protein and how far the protomol extends outside the cavity, respectively. The proto_thresh was selected as 0.5 and proto_bloat was selected as 0. After the preparation of the protomol, molecular docking was performed. All molecules designed were docked to the prepared structure of PDE9A and 20 molecules with the highest docking scores and appropriate binding modes went into the following MD simulations and binding free energy calculations.

### Molecular dynamics simulation

The MM-PBSA approach^36–38^ was applied for the binding-free-energy calculations using the 100 snapshots isolated from the final 1.0 ns period of each 8 ns MD simulations trajectory. The electrostatic contribution to the solvation-free energy was determined using the PBSA program in the Amber 16 suite^39^. A grid size of 0.5 Å was used. The interior and exterior dielectric constants for the solute and solvent were set to 1 and 80, respectively. The optimised atomic radii set in Amber 16 were used. To speed up calculations without much decrease in accuracy, the entropy contributions are omitted.

## Result and discussion

### The design of novel PDE9A inhibitors with assistance of molecular docking and dynamics simulation

**Figure 1.**
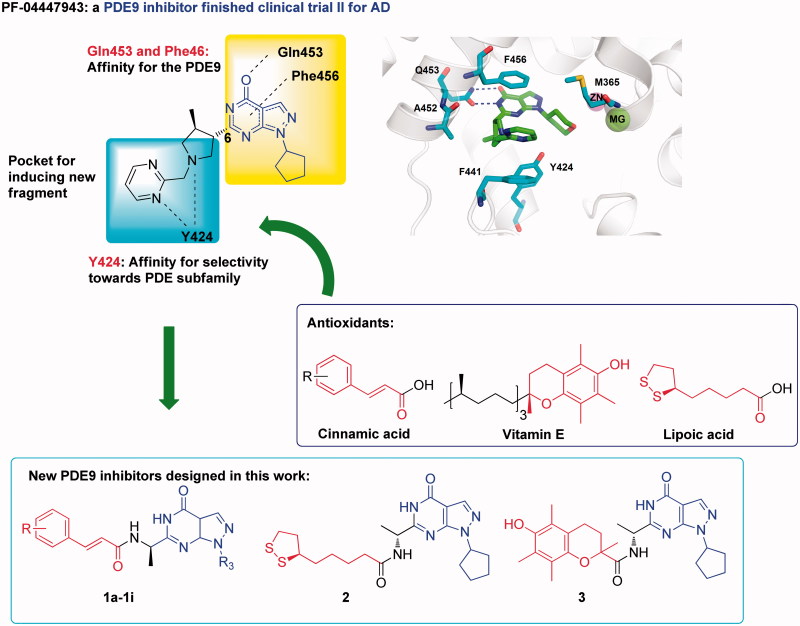
The design of PDE9 inhibitors with antioxidant activities.

The crystal structures of PDE9A-inhibitors showed that two conserved residues Gln453 and Phe456 play essential roles for binding of the inhibitors^11^^,^^18^^,^^27^^,^^34^ (Figure 1). Most PDE9A inhibitors contain a pyrazolopyrimidinone scaffold to form hydrogen bond with Gln453 and π–π interactions with Phe456. A narrow and long pocket next to pyrazolopyrimidinone is available in the PDE9 structure for introducing an extra fragment with antioxidant activity. In addition, Tyr424 in this narrow pocket, which is unique to PDE8 and PDE9, has been shown to be important for selectivity of PDE9 inhibitors over other PDEs^27^^,^^34^. Thus, we took pyrazolopyrimidinone as the scaffold of our designed compounds. Functional fragments from common antioxidants such as ferulic acid, caffeic acid, lipolic acid were attached to 6-position of pyrazolopyrimidinone with various linkers for antioxidant activities (compounds structure shown in Figure S1)^28^^,^^40^^,^^41^.

In order to improve the efficiency and reduce synthetic work, molecular docking was performed on the designed compounds to predict their binding patterns. This is quite different from the usual drug discovery in which molecular docking was used to predict the binding mode after lead compounds were identified. Furthermore, binding free energies between designed compounds and protein in atomic scales, which can improve the hit rate significantly but are not available in the experiments sometimes were obtained by the molecular dynamics simulation for a more precise prediction. According to the calculation results, we found that the compounds with an amide group as the linker had stable MD simulations trajectories and high binding free energies (compounds structure in Figure 1, Binding free energies in Table 1, Binding mode in SP), indicating that they might be potential PDE9A inhibitors.

**Table 1.** The Inhibitory activities against PDE9A, binding free energies, and oxygen radical absorbance capacity of compounds **1a**–**1l**, **2**, and **3**.


Compound	R	IC_50_±SD (nM)^a^	GBTOT (kcal·mol^−1)^	ORAC
**1a**		126 ± 15	−31.09 ± 2.64	2.4
**1b**		64 ± 2	−29.78 ± 2.33	2.5
**1c**		65 ± 4	−29.53 ± 2.82	0.23
**1d**		83 ± 11	−35.41 ± 3.09	1.26
**1e**		>200	−35.77 ± 2.64	0.50
**1f**		190 ± 7	−27.39 ± 3.05	0.12
**1g**		63 ± 3	−35.71 ± 2.81	2.9
**1h**		56 ± 7	−38.16 ± 3.08	3.3
**1i**		133 ± 22	−33.80 ± 3.08	1.00
**1j**		186 ± 7	−35.52 ± 2.72	0.08
**1k**		130 ± 6	−43.39 ± 2.53	0.02
**1l**		57 ± 4	−28.79 ± 2.93	2.6
**2**		25 ± 4	−40.01 ± 3.74	0.28
**3**		>500	−31.98 ± 2.52	0.24
BAY73-6691	–	46	–	n.a.^c^
Ferulic acid	–	n.d.^b^	–	1.6

a IC_50_ values are given as the means of three independent determinations.

b n.d. means not determined.

c n.a. means not active.

In order to filter possible false hits, pan-assay interfering compound substructures (PAINS) screening was performed using the online program PAINS-Remover (http://cbligand.org/PAINS)^42^. All the compounds with an amide group as the linker (Figure 1) passed this test and were then synthesised.

### Chemistry

**Scheme 1.**
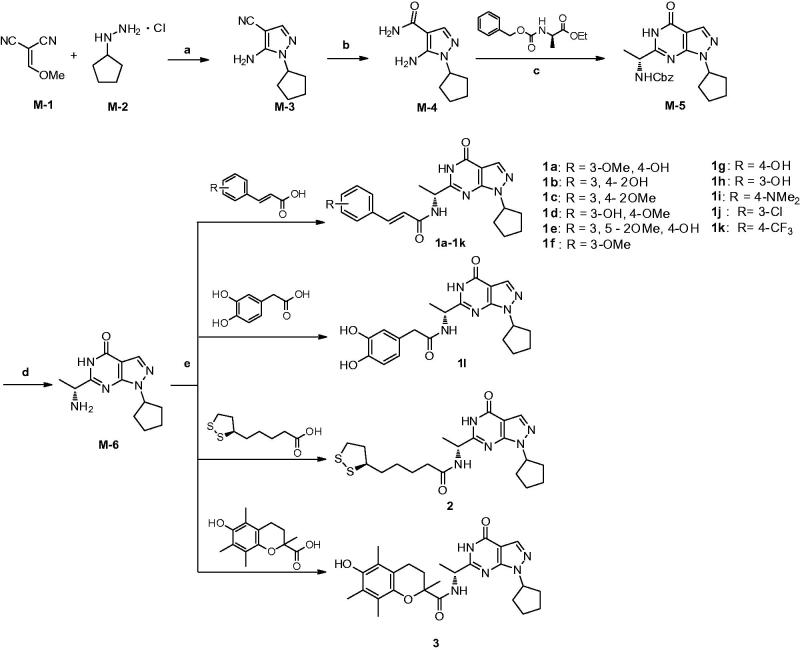
Reagents and conditions: (a) Triethylamine, 0 °C, r.t., 3 h, then reflux 3 h, EtOH; (b) NH_3_·H_2_O,H_2_O_2_, EtOH, r.t.; (c) (*R*)-ethyl-2-(((benzyloxy)carbonyl)amino)propanoate, 60% NaH, dry THF, r.t.; (d) Pd/C, MeOH, H_2,_ r.t.; (e) EDC·HCl, DMAP, DMF, r.t.

As outlined in Scheme 1, condensation of 2-(methoxymethylene)malononitrile (**M-1**) with cyclopentylhydrazine (**M-2**) afforded **M-3** in the presence of triethylamine. Hydrolysis of the cyano group of **M-3** in the presence of ammonium hydroxide and hydrogen peroxide produced **M-4** with an amide group^27^. **M-5** was synthesised by the reaction of **M-4** with (*R*)-ethyl-2-(((benzyloxy)carbonyl)amino)propanoate under basic condition at room temperature. The benzoyl group of **M-5** was removed by hydrogenation in hydrogen atmosphere and the presence of the Pd/C catalyst, producing **M-6** in high yield^20^. Compounds **1a-1l** were prepared in moderate to high yield by condensation reaction of **M-6** with the acids such as ferulic acid^28^. Compounds **2** and **3** were prepared in the same procedure by using lipoic acid and trolox^28^.

### Inhibition of designed compounds on PDE9A *in vitro*

The inhibitory activities of compounds **1a–1l, 2, and 3** against PDE9A were assayed with the procedure previously reported^26^. The results are summarised in Table 1. **BAY73–6691,** a PDE9 inhibitor developed by Bayer company serves as the reference in the assay (Lit: IC_50_=55nM)^43^.

Compounds **1a–1l**, which contain the fragment of ferulic acid derivatives, have the IC_50_ values of 56–190 nM against PDE9A. The substitutions on the phenyl ring showed remarkable impacts on the inhibitory activities against PDE9A. Compounds (**1a, 1b, 1d, 1g, 1h**) with a hydroxyl group on the phenyl ring have better inhibitory activities than other substitutions. Among all the compounds in series **1**, compound **1h** with the 3-hydroxyl on the phenyl ring gave the best IC_50_ value of 56 nM, which is as effective as **BAY73–6691** (55 nM). Compounds **1b** with two hydroxyl group and **1c** with two methoxy groups gave comparable inhibitory activities. Introduction of an additional methoxy group from compound **1a** to **1e** makes the IC50 drop to >200 nM, suggesting steric hinder in the binding pocket is important. Using other substitution groups such as dimethylamine, trifluoromethyl, and chloro to instead the methoxy or hydroxyl group of ferulic acid formed compounds **1i–1k**. The inhibitory activities of these compounds against PDE9A dropped to 130–180nM. We concluded that the hydroxyl group on the phenyl ring might form interactions with residues in the binding pocket of PDE9A, improving the inhibitory activity against PDE9A. Compared with **1b** derived from caffeic acid, compound **1l** which does not contain a double bond, gave a comparable IC_50_ value of 57nM, suggesting that the double bond may not be essential for the affinity with PDE9A. Compound **2** having lipolic acid linked to 6-position of pyrazolopyrimidinone gave the best IC_50_ of 25 nM, in comparison with IC_50_>500 nM of compound **3** with trolox group. We speculated that the trolox group might be too large to fit into the binding pocket of PDE9A. The linear correlation between docking score values and IC_50_ values of compound **1a–1l**, **2**, and **3** was observed with a moderate *R*_2_ value of 0.58 (Table S1 and Figure S2). Compound **3** and **1e** with the lowest docking score values, gave the lowest inhibitory activities against PDE9A. However, for some compounds such as **1i** and **1f**, there are some deviations between the docking score values and IC_50_ values.

### Antioxidant activity test by the ORAC method

Oxygen radical absorbance capacity assay (ORAC-FL) has been widely used to evaluate antioxidant activities of hydrophilic antioxidants including ferulic acid and phenolic derivatives. Thus, the antioxidant activities of compounds **1a–1l** and compound **2** were evaluated by ORAC-FL method^31^^,^^32^. The results were expressed as trolox equivalents and summarised in Table 1. Ferulic acid was used as the positive reference compound, showing an ORAC-FL value of 1.6 trolox equivalents. The feruic acid derivatives with the hydroxyl group on the phenyl ring (**1a, 1b, 1d–1h, 1l)** showed good ORAC-FL values in a range of 1.3–3.3 fold of trolox equivalents, while compounds without the hydroxyl group (**1c, 1f, 1j, and 1k)** showed low ORAC-FL values except for compound **1i** with dimethylamino group substituted. These results are consistent with the previous reports that the hydroxyl-phenyl groups of the ferulic acid are the main source of antioxidant activity^41^. To our surprise, compound **2** with the fragment of lipolic acid showed low antioxidant activity.

### Selectivity of compounds 1h and 2 over PDE1 and PDE8

The PDE super-family of enzymes are divided into 11 subfamilies according to the differences in structure and distribution. Each PDE subfamily is in charge of different biological process. In order to reduce drug adverse effects, the selectivity over other PDEs is important for potential PDE inhibitors. Since the binding pocket of PDE subfamily is structurally similar, selective PDE inhibitors are not easy to be obtained. For the PDE9A inhibitors, the selectivity over PDE1 and PDE8 are usually difficult to obtain^18^^,^^20^^,^^34^. Thus, compound **1h** with PDE9A inhibition and good antioxidant activity, compound **2** with the best inhibition towards PDE9A were chosen for exploration of selectivity over PDE1 and PDE8 (Table 2). Compounds **1h** and **2** at 1 μM inhibited 13 and 59% activity of PDE1B, thus estimating 25 and 40-fold selectivity, respectively. The selectivity is better than **BAY73-6691** but not **PF-04447943**^18^^,^^43^. The inhibitory activities of compounds **1h** and **2** against PDE8A at 10 μM were 10 and 9%, respectively, implying the 250–400 fold selectivity of these compounds towards PDE8A. Thus, both compounds **1h** and **2** are good selective PDE9 inhibitors.

**Table 2.** Inhibitory activities of compounds **1h** and **2** with PDE1 and PDE8.

Compound	IC_50_ (nM) of PDE9A	Inhibition of PDE1B at 1000 nM	Inhibition of PDE8A at 10 μM
**1h**	56 ± 1	13% (>25)^a^	10% (>250)^a^
**2**	25 ± 4	59% (40)^a^	9% (>400)^a^
**BAY73-6691**	55^b^	1400 (25)^b^	–
**PF-04447943**	8^c^	1394 (174)^c^	–

a The numbers in parentheses are the fold of selectivity of inhibitors against PDE9 over other PDEs (IC_50_, nM).

b Data was obtained from the reported literature^43^.

c Data was obtained from the reported literature^18^.

### Effects of compounds 1h and 2 on cell viability

**Figure 2.**
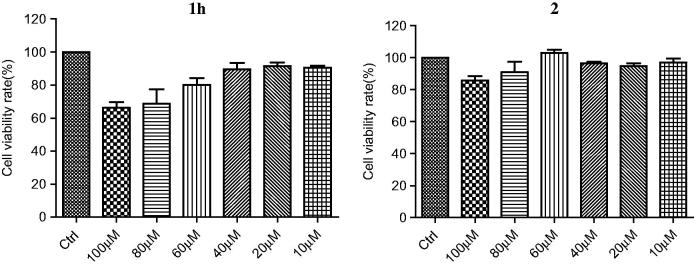
Cell viability of compounds **1h** and **2**.

The cytotoxicity of compounds **1h** and **2** were tested using human neuroblastoma cell line SH-SY5Y by the MTT method^33^. As shown in Figure 2, after incubation for 48 h, the cellular viability was about 90% at 40 μM of compound **1 h** and 70% at 100 μM. Compound **2** at 100 μM showed no significant effect on cell viability. Control experiments were performed, eliminating the possibility that compounds **1h** and **2** interfered with the MTT assay (Figure S8). These results suggested that both **1h** and **2** are not toxic to SH-SY5Y cells and potential candidates for further development as therapeutics for AD.

### Analysis of the structure-activity relationship by molecular docking and dynamics simulation

In order to improve the efficiency and reduce synthetic work in the process of identifying PDE9 inhibitors, the binding mode, and binding free energies of all these compounds were performed by molecular docking and dynamics simulation.

**Figure 3.**
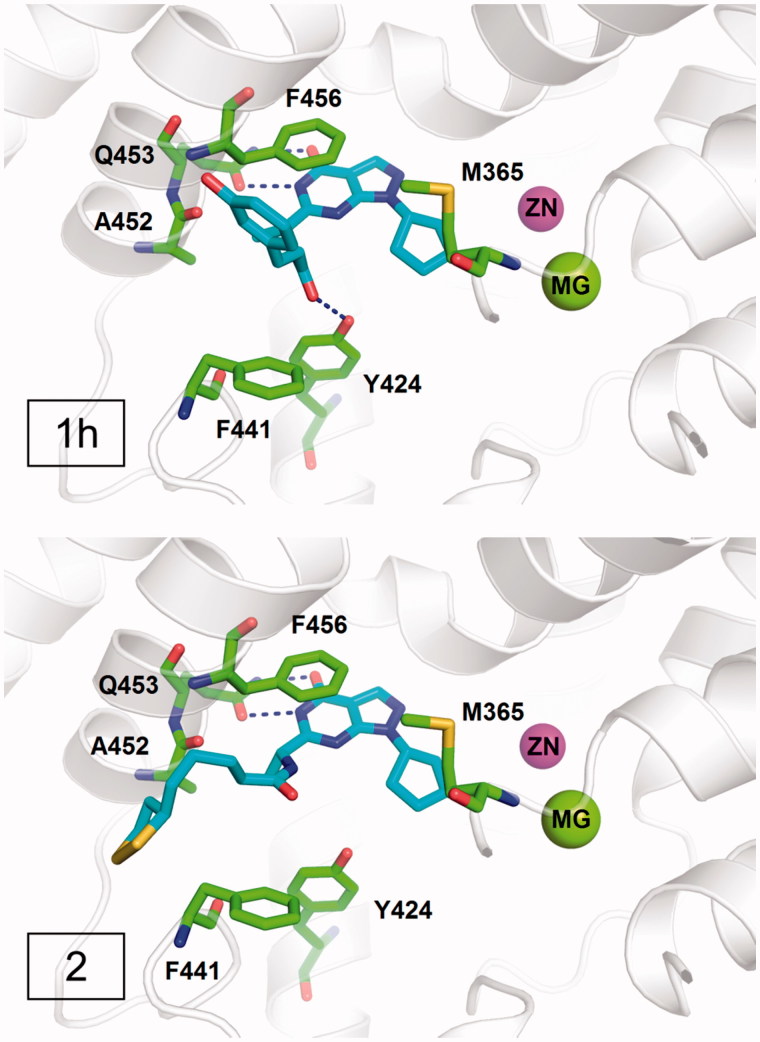
The binding modes of compounds **1h** and **2**.

According to the difference in the binding modes, the designed compounds in series 1 can be divided into three classes. Compounds **1a, 1c, 1d,** and **1e** adopted a similar binding mode (Figure S3) and form two hydrogen bonds with invariant Gln453 and π–π interactions with conserved Phe456. Compounds **1b, 1j,** and **1k** formed an additional hydrogen bond with Ala452 in addition to the interactions with Gln453 and Phe456 (Figure S4). Compounds **1f, 1g, 1h,** and **1i** formed an additional hydrogen bond with Tyr424 besides the interactions with Gln453 and Phe456 (Figure S5). As is mentioned before, the interactions with Gln453 and Phe456 are the main reasons for the affinity of inhibitors against PDE9. Thus, all these compounds should have good inhibitory activities against PDE9A. Further biological tests demonstrated that most compounds exhibited the inhibition activity against PDE9 with IC_50_ below 200 nM except compound **1e**. Compound **1h**, which has IC_50_ of 57 nM against PDE9 and the highest ORAC value of 3.3 trolox, belongs to the class II, forming an additional hydrogen bond with Tyr424 (Figure 3). Since Tyr424 is unique to PDE9 and PDE8, the interaction with Tyr424 will render high selectivity of PDE9 inhibitors. This is supported by the good selectivity of compound **1h** over PDE1B and PDE8A. Compound **2**, which has IC_50_ of 25 nM against PDE9 and good selectivity over PDE1 and PDE8 showed no interaction with Tyr424, implying a new way to design selective PDE9 inhibitors (Figure 3).

The binding free energy calculations were performed by the MM-PBSA method. The results of compounds **1h** and **2** are shown in Table 3, suggesting that van der Waals(*Δ*E_vdw_) and electrostatic components (*Δ*E_ele_) are two important factors for the high affinity of both compounds **1h** and **2** while polar component to solvation (*Δ*E_pol,solv_) is less important. 

**Table 3.** Components of the binding free energy (kcal/mol) for PDE9A in complex with **1h** or **2** by using the MM–PBSA method.

Compound	**1h**	**2**
*Δ*E_vdw_	−50.32 ± 3.38	−50.86 ± 3.14
*Δ*E_ele_	−33.66 ± 5.38	−25.46 ± 3.95
*Δ*E_pol,solv_	50.40 ± 4.08	41.05 ± 2.28
*Δ*E_nonpol,solv_	−4.57 ± 0.10	–4.74 ± 0.13
*Δ*G_gas_	−83.99 ± 5.79	−76.32 ± 4.04
*Δ*G_solv_	45.83 ± 4.05	36.31 ± 2.30
*Δ*G_bind_	38.16 ± 3.08	−40.01 ± 3.74

MM–PBSA: molecular mechanical and Poisson–Boltzmann/surface accessible; ΔE_ele_: electrostatic interactions calculated by the MM force field; ΔE_vdw_: van der Waals’ contributions from MM; ΔE_nonpol, sol_: the nonpolar contribution to solvation; ΔE_ele, sol_: the polar contribution to solvation; T*Δ*S: entropic contribution by using N-mode methods; ΔE: the predicted binding free energies with entropic contribution omitted; ΔG_bind, pred_: the predicted binding free energies with entropic contribution.

## Conclusion

In summary, a series of novel pyrazolopyrimidinone derivatives have been designed, synthesised and evaluated as multifunctional anti-AD ligands, in combination of PDE9A inhibition and antioxidant activities. With the assistance of molecular docking and dynamics simulation, high selective PDE9 inhibitors with antioxidant activities have been successfully developed as shown by compounds **1h** (IC_50_ of 56 and 3.3 trolox of ORAC). These PDE9 inhibitors do not disturb cell viability of SH-SY5Y and thus are worth for further development as therapeutics for treatment of AD. The structure-activity relationship was explained by the binding modes of inhibitors with PDE9 protein, providing evidence for further structural modification.

## Supplementary Material

IENZ_1412315_Supplementary_Material.pdf
