# Correction to: Zhang et al., Discovery of novel PDE9A inhibitors with antioxidant activities for treatment of Alzheimer’s disease

**DOI:** 10.1080/14756366.2018.1427691

**Published:** 2018-01-24

Zhang C, Zhou Q, Wu X-N, Huang Y-D, Zhou J, Lai Z, Wu Y, and Luo H-B. (2017). Discovery of novel PDE9A inhibitors with antioxidant activities for treatment of Alzheimer’s disease. *Journal of Enzyme Inhibition and Medicinal Chemistry*. 33:260–70. https://doi.org/10.1080/14756366.2017.1412315.

When the above article was first published online, the name of one co-author was incorrectly written as Yat-Sen Huang. This has now been corrected in the online version as ‘Ya-Dan Huang’.

Taylor & Francis apologises for this error.

