# Supplementary material for: Discovery of novel PDE9A inhibitors with antioxidant activities for treatment of Alzheimer’s disease
Source: J Enzyme Inhib Med Chem. 2017 Dec 22;33(1):260–70. doi: 10.1080/14756366.2017.1412315 (PMC7011943; doi:10.1080/14756366.2017.1412315)

Supporting Information

Discovery of novel PDE9A inhibitors with antioxidant activity for treatment of Alzheimer's disease

Chen Zhang[a, †], Qian Zhou[a, †], Xu-Nian Wu[a,], Ya-Dan Huang[a,], Jie Zhou[a,], Zengwei Lai[a,*], Yinuo Wu[a,*] and Hai-Bin Luo [a, b]

[a] School of Pharmaceutical Sciences, Sun Yat-Sen University, Guangzhou 510006, PR China

[b] Collaborative Innovation Center of High Performance Computing, National University of Defense Technology, Changsha 410073, China

[c] State Key Laboratory for Chemistry and Molecular Engineering of Medicinal Resources, Guangxi Normal University, 15 Yu Cai Road, Guilin 541004, China

† These authors contribute equally.

*Corresponding authors: wyinuo3@mail.sysu.edu.cn (Y. Wu). or 2860879@sina.com (Z. Lai)

Content

1. Figure S1-S8 and Table S1 --S2

2. ^{1}HNMR and ^{13}CNMR of compounds **1a-1l, 2** and **3** ---S9

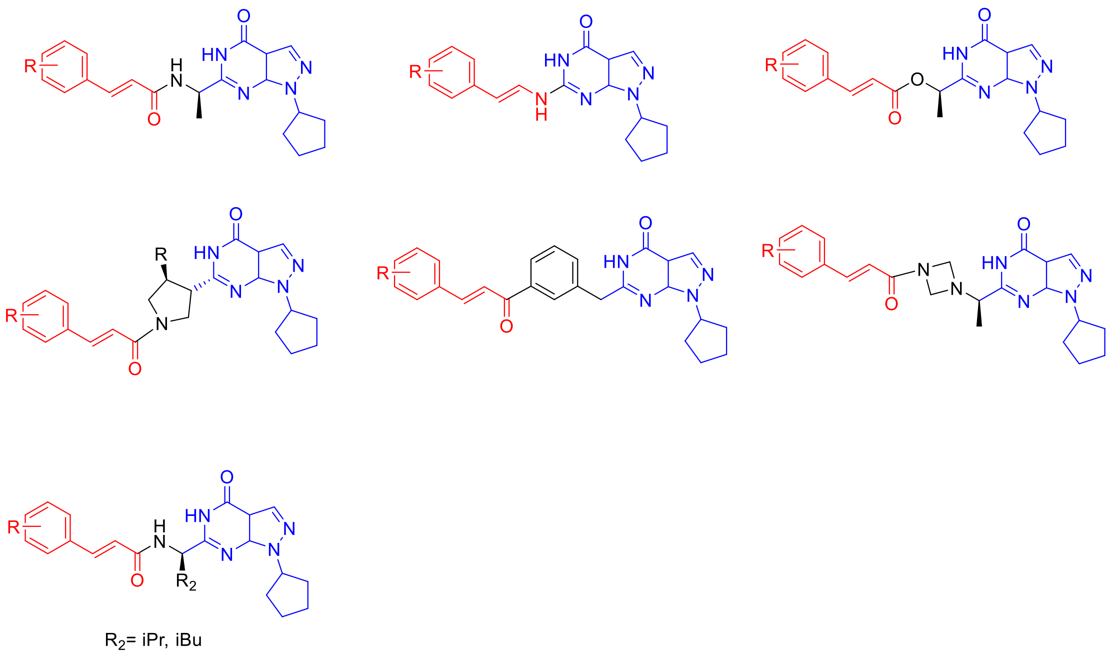

Figure S1: Designed compounds with different linkers.

Table S1. The Docking Score values and IC_{50} of compounds **1a-1l**, **2**, and **3**.

Compound	Docking Score[a]	IC_{50}± SD (nM)[b]
**3**	3.7284	>500[c]
**1e**	5.6790	>200[c]
**1a**	5.8646	126 ± 15
**1l**	6.2419	57±4
**1k**	6.3890	130±6
**1d**	6.7393	83± 11
**1b**	6.9738	64± 2
**1j**	7.0099	186±7
**1h**	7.1075	56±7
**2**	7.1897	25±4
**1f**	7.2400	190±7
**1c**	7.2997	65± 4
**1i**	7.8117	133±22
**1g**	8.6352	63± 3

[a] Docking Score values are given by the total_score of Surflex-Dock method embedded in Tripos Sybyl 2.0.

[b] IC_{50} values are given as the mean of three independent determinations.

[c] The IC_{50} values of **1e** and **3** are treated as 200 and 500 nM for the correlation with Docking Score values.

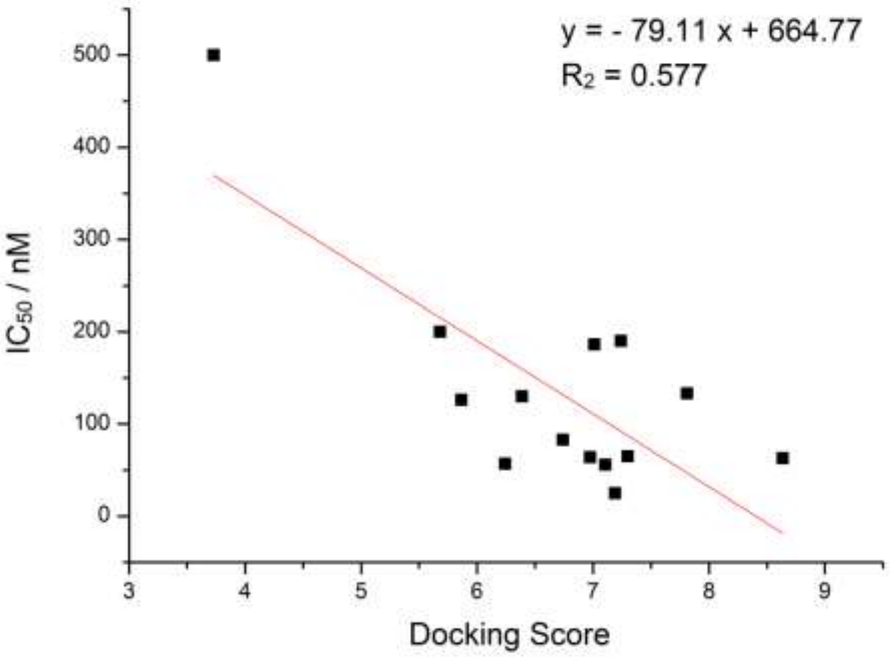

Fig. S2. The correlation between Docking Score values and IC_{50} values of compound **1a-1l**, **2**, and **3**.

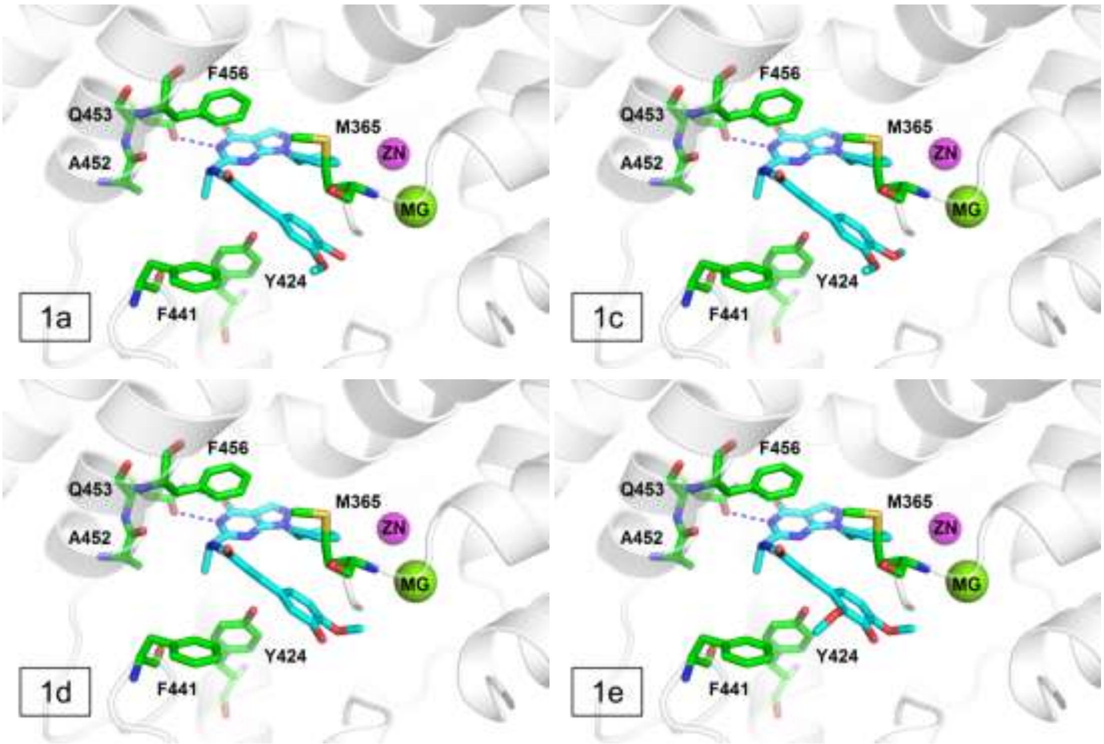

Figure S3: Binding modes of compound **1a, 1c, 1d** and **1e.**

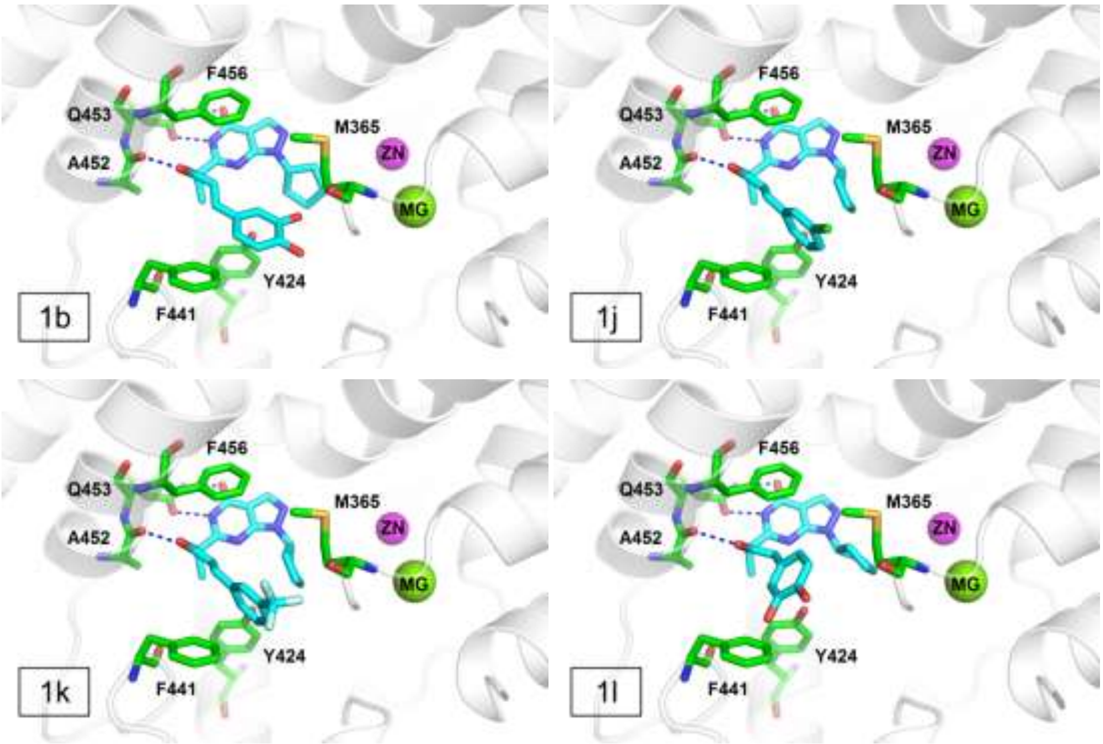

Figure S4: Binding modes of compounds **1b, 1j, 1k** and **1l**.

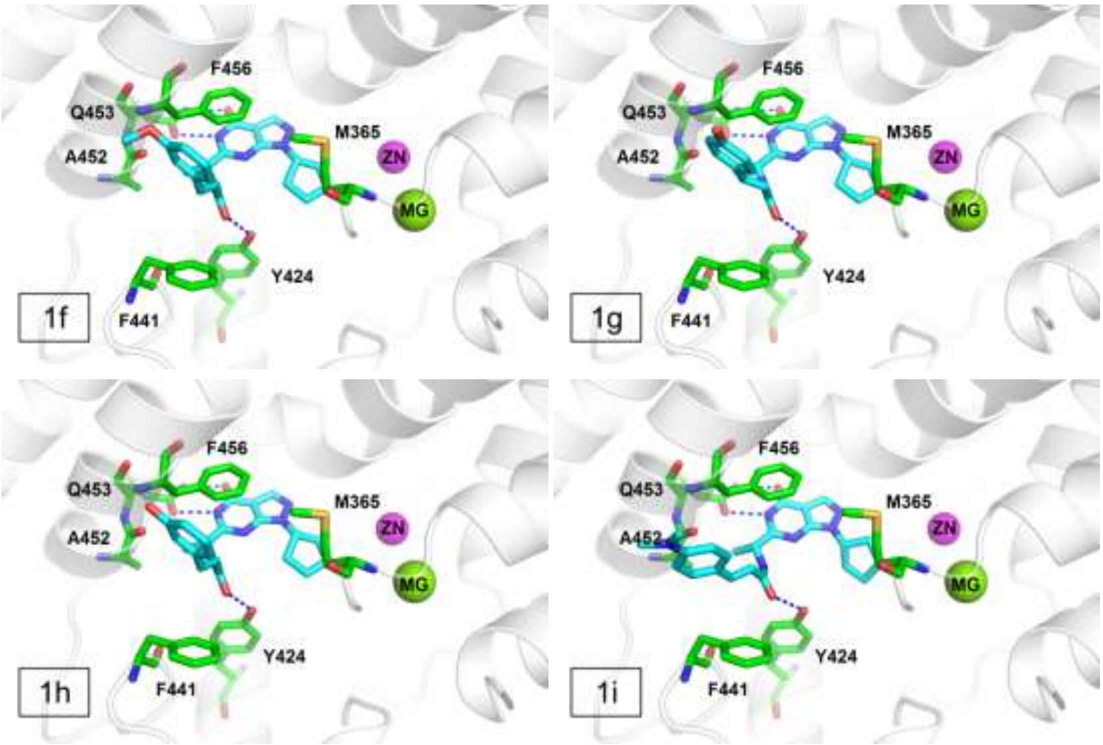

Figure S5: Binding modes of compounds **1f, 1g, 1h** and **1i.**

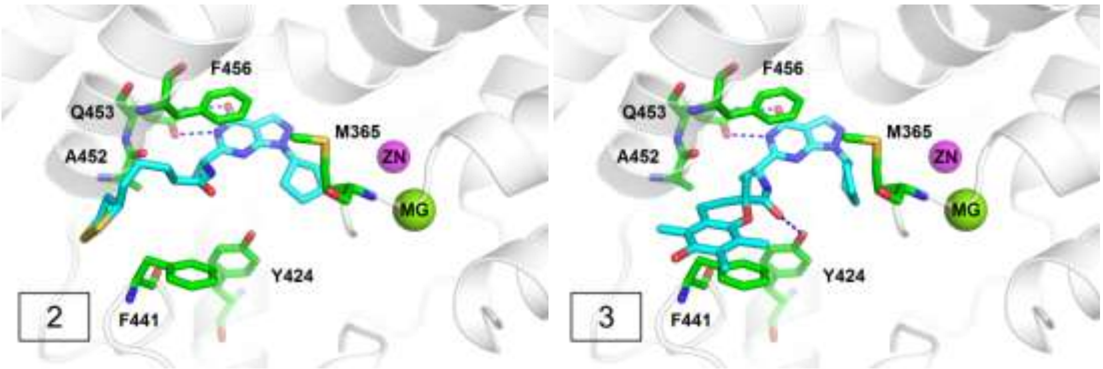

Figure S6: Binding modes of compound**s 2 and 3.**

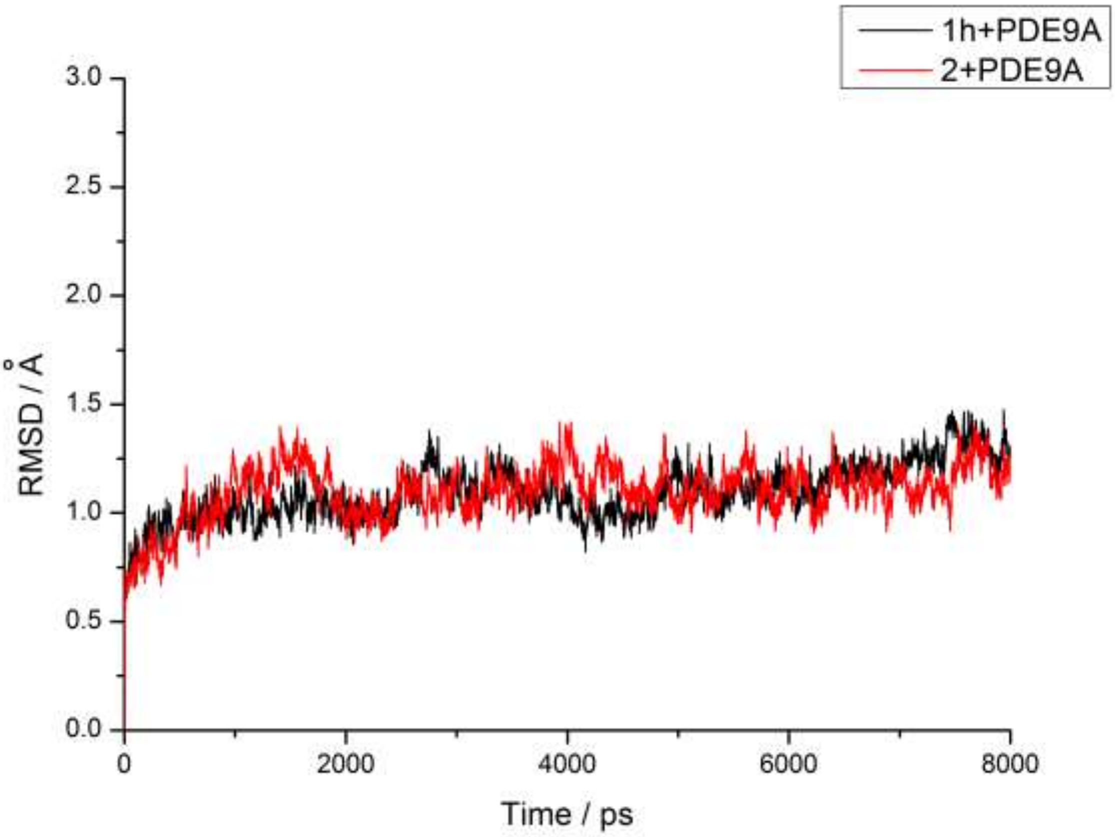

Figure S7: The RMSD plots of compounds **1h** and **2.**

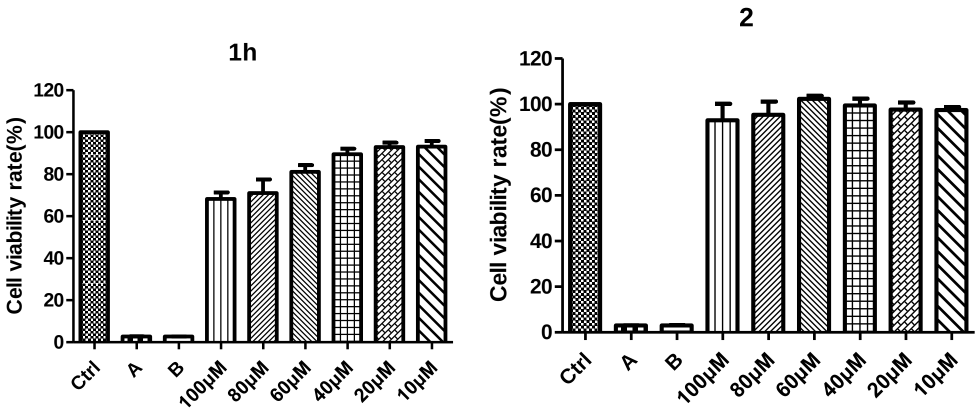

Figure S8: Cell viability of compounds **1h** and **2.** A) without cell and compounds A) without cell but compounds were added at 100μM

^{1}H and ^{13}C NMR of **M-6**

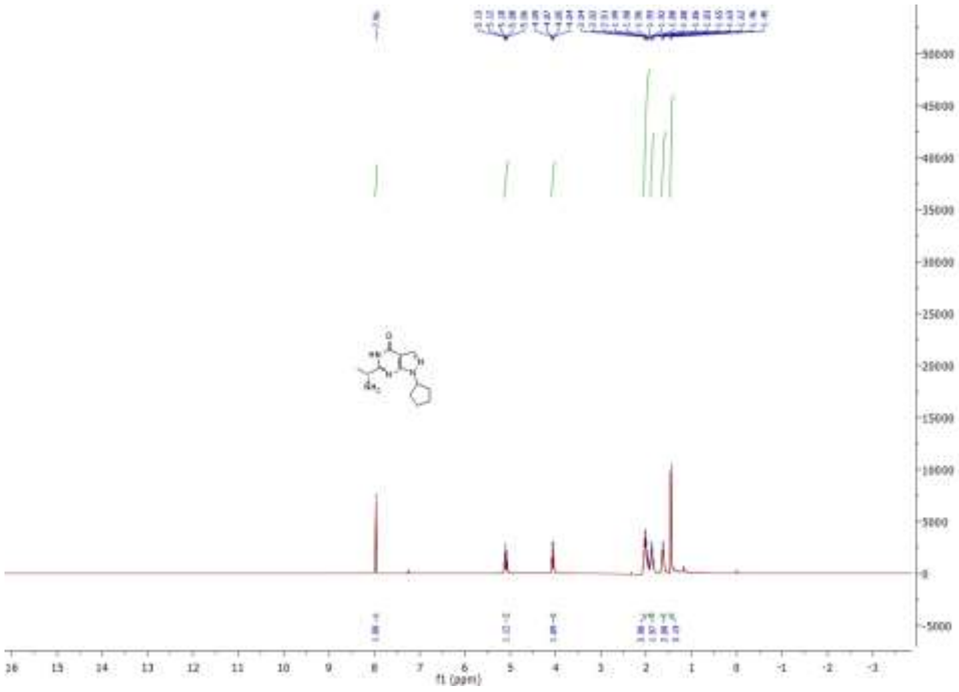

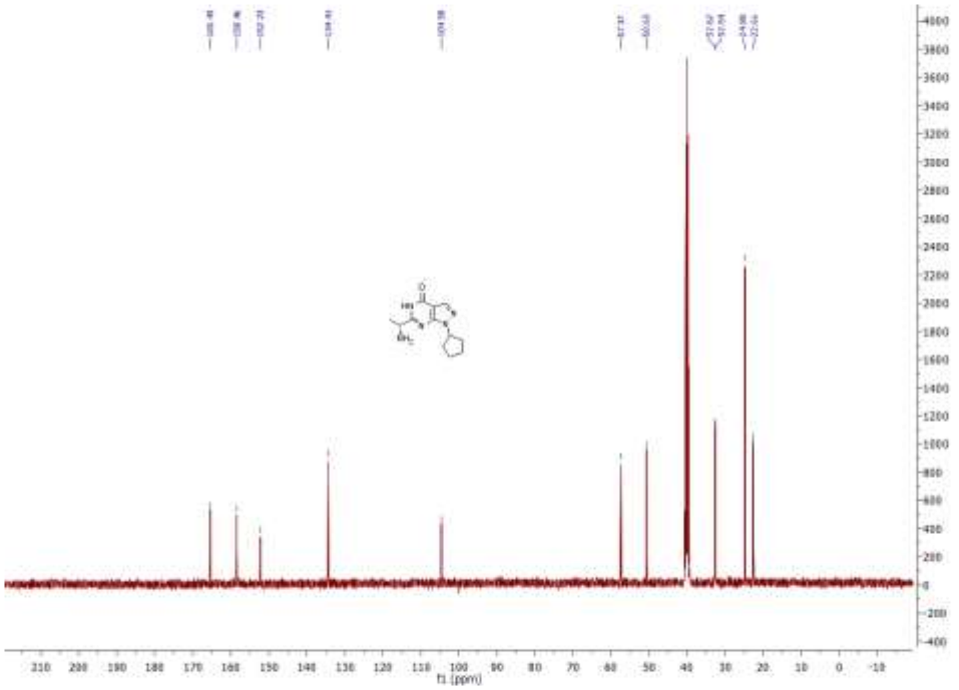

^1H and ^{13}C NMR of **1a**

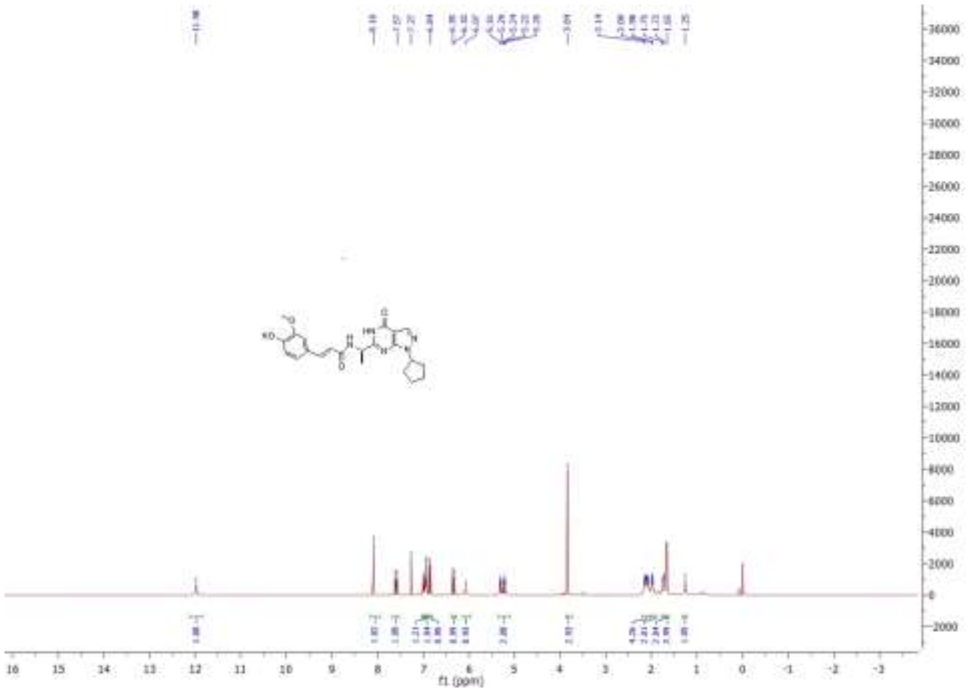

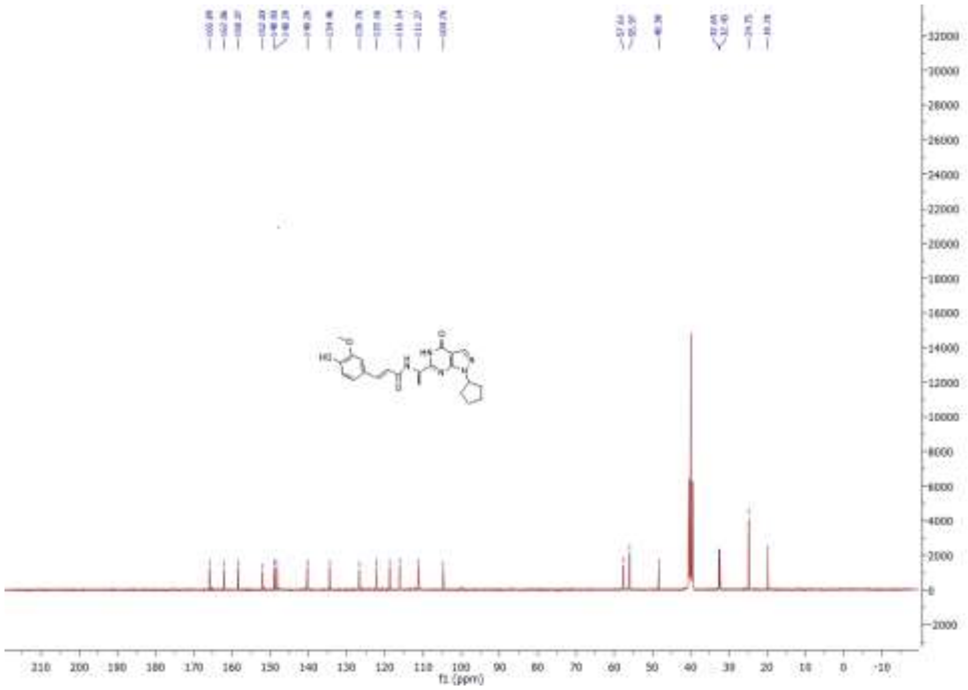

^{1}H and ^{13}C NMR of **1b**

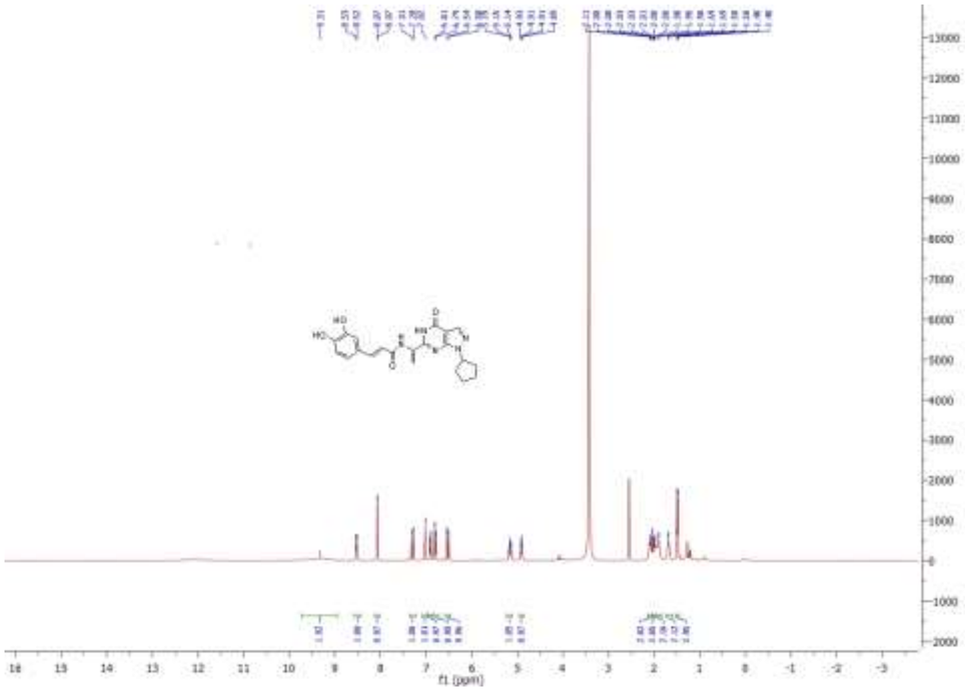

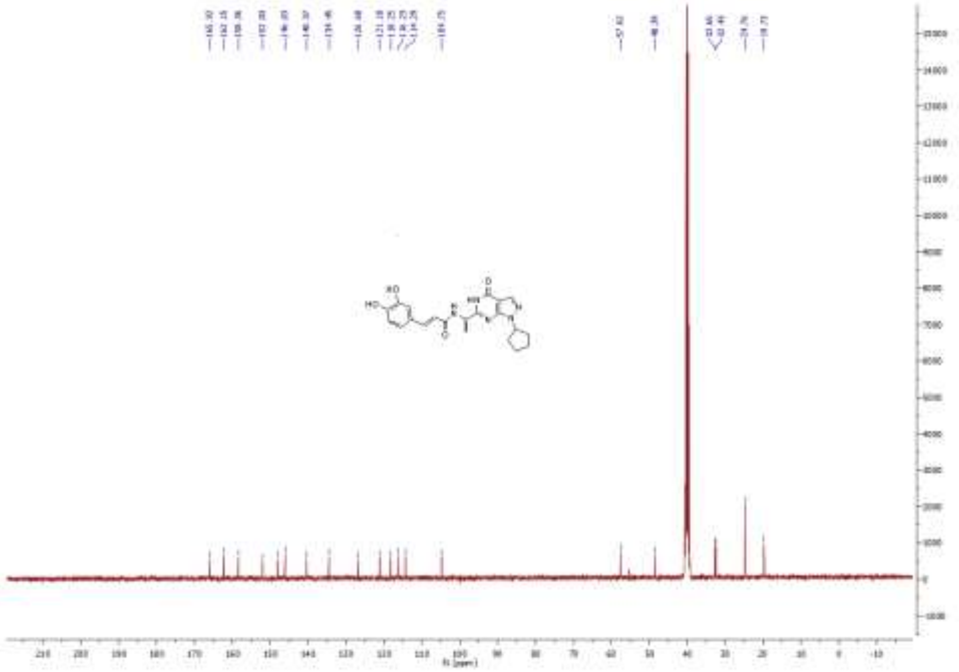

^{1}H and ^{13}C NMR of **1c**

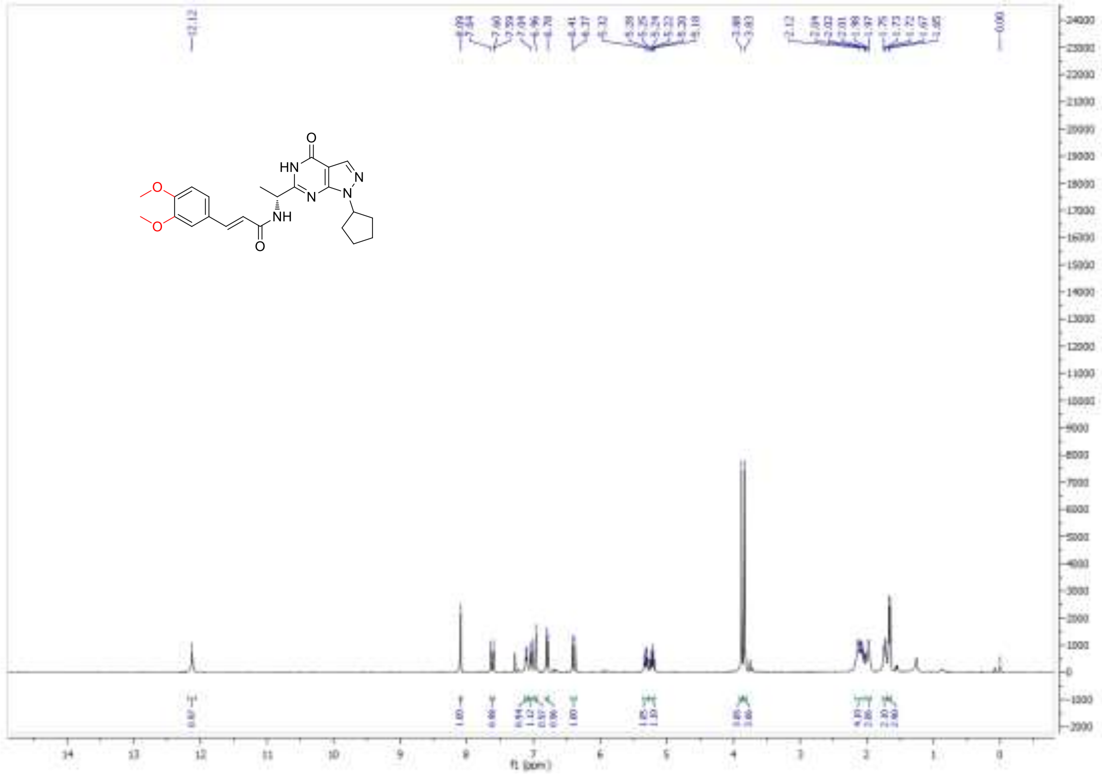

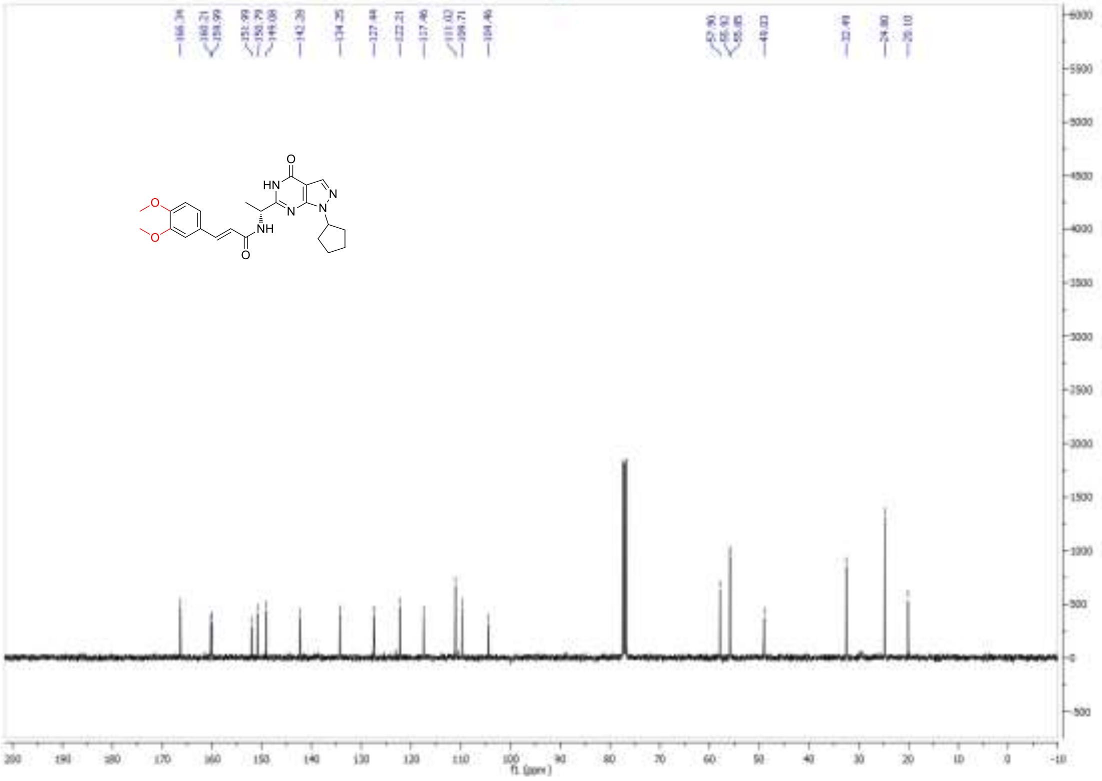

^{1}H and ^{13}C NMR of **1d**

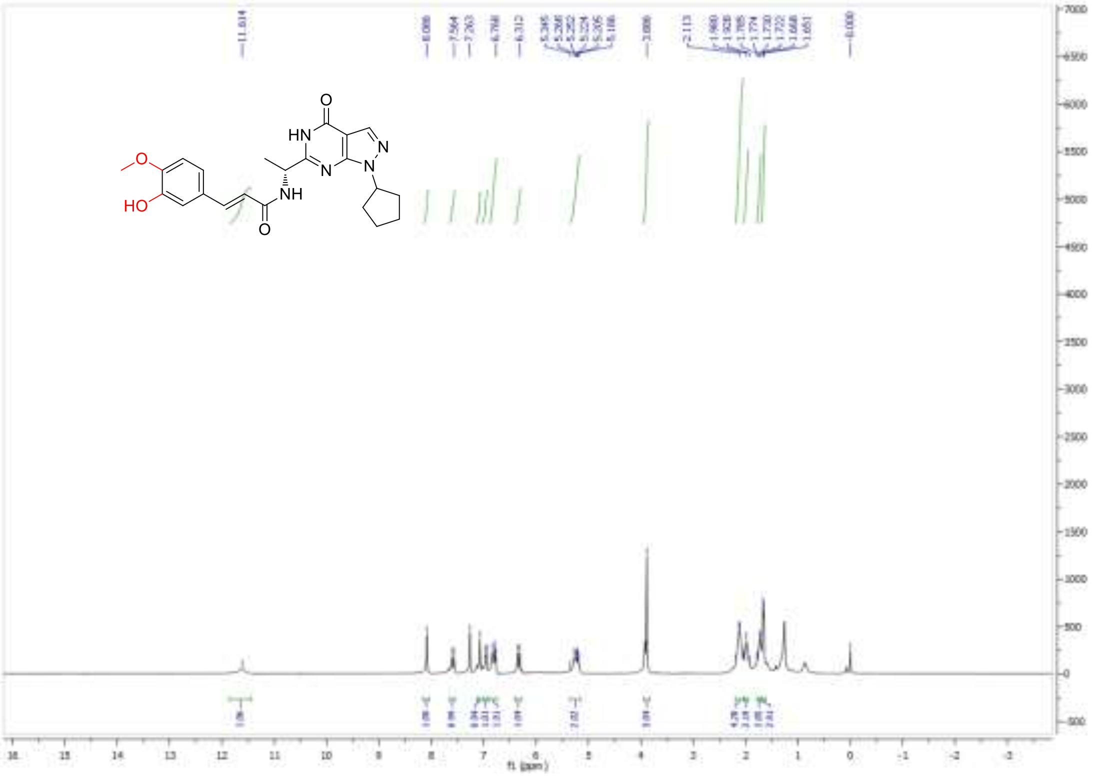

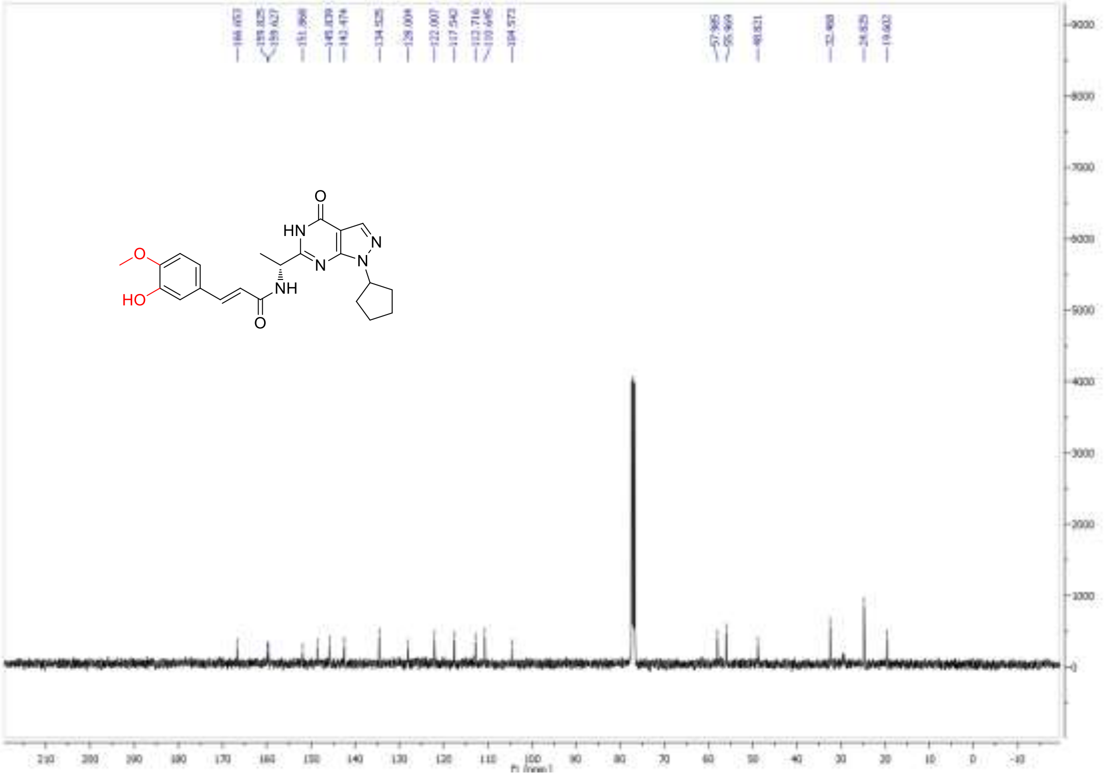

^{1}H and ^{13}C NMR of **1e**

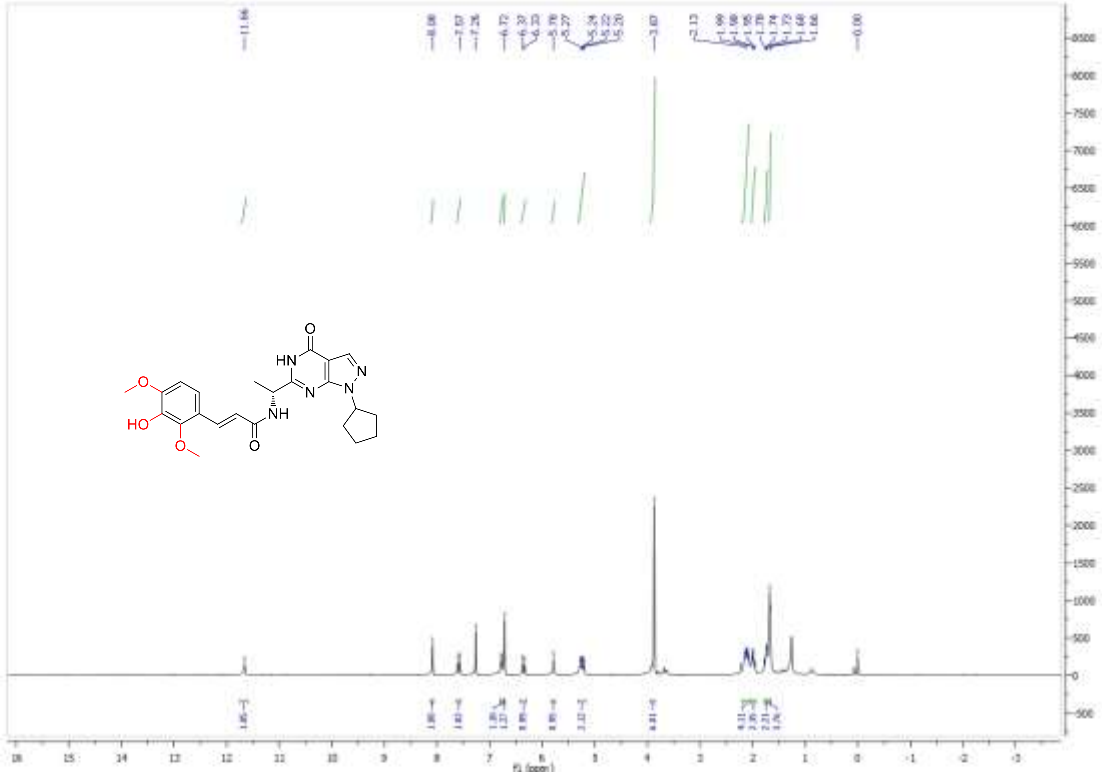

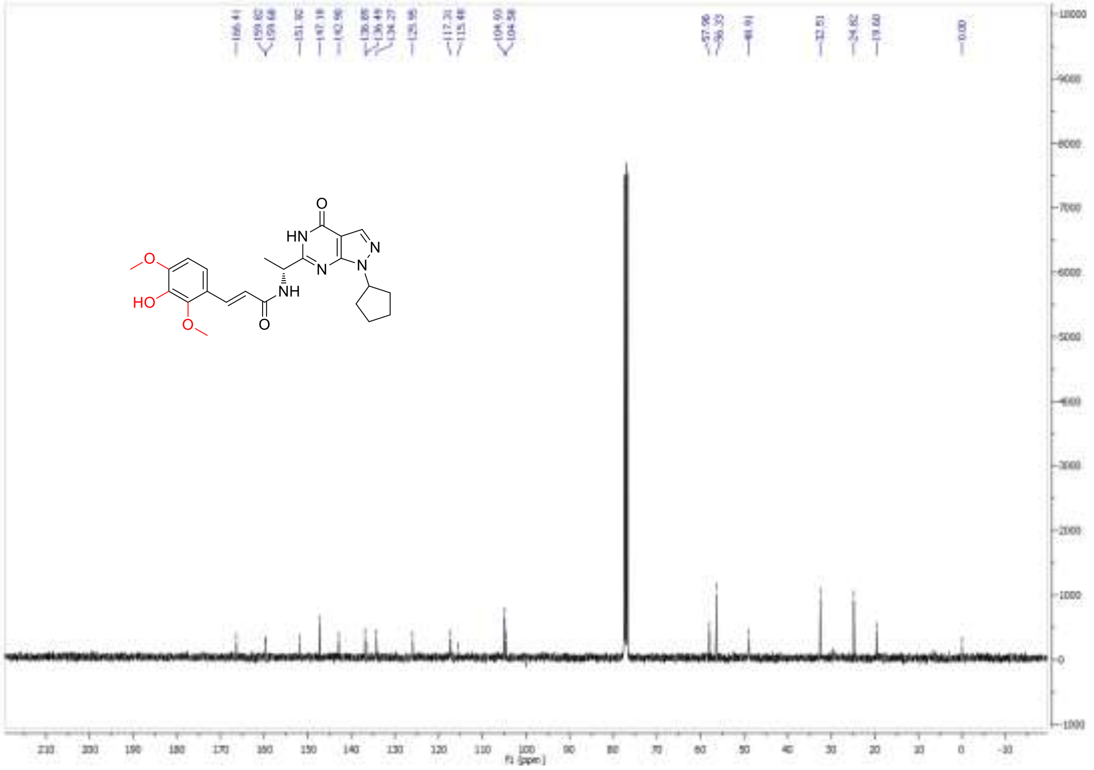

^{1}H and ^{13}C NMR of **1f**

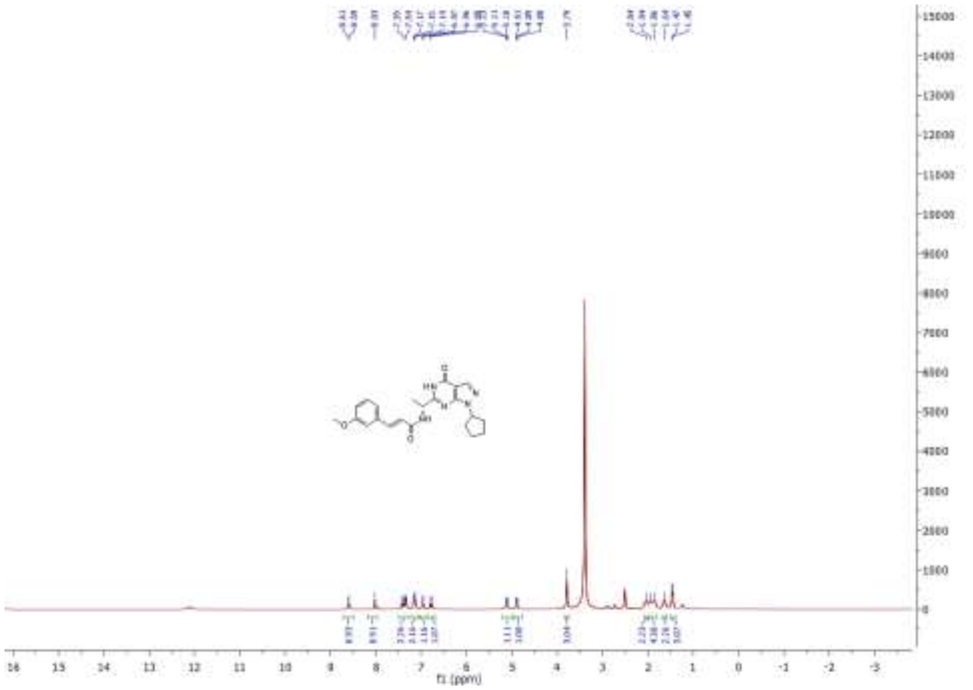

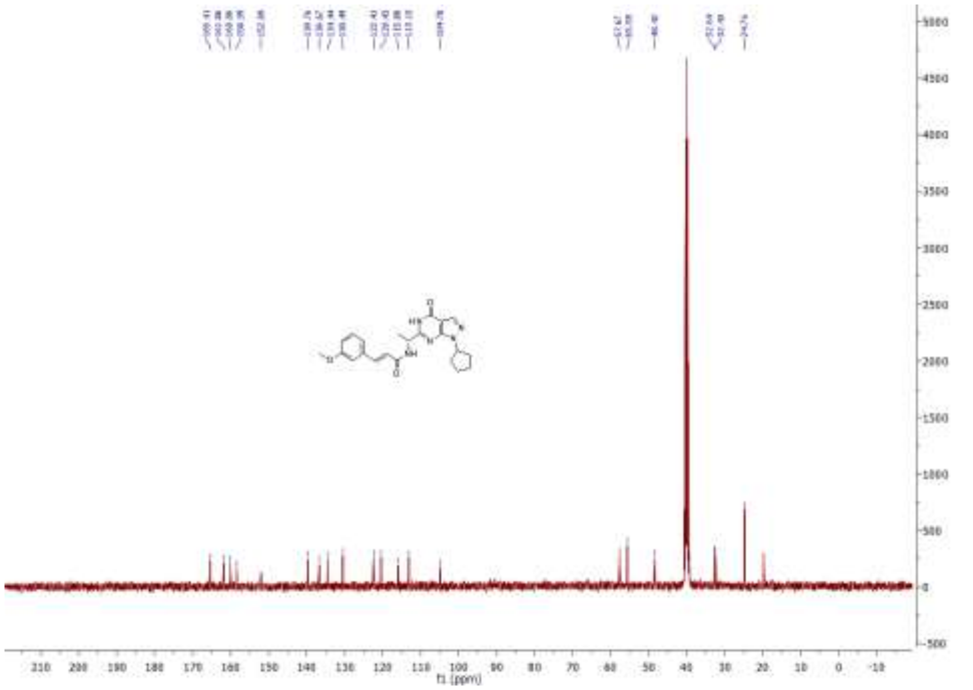

^{1}H and ^{13}C NMR of **1g**

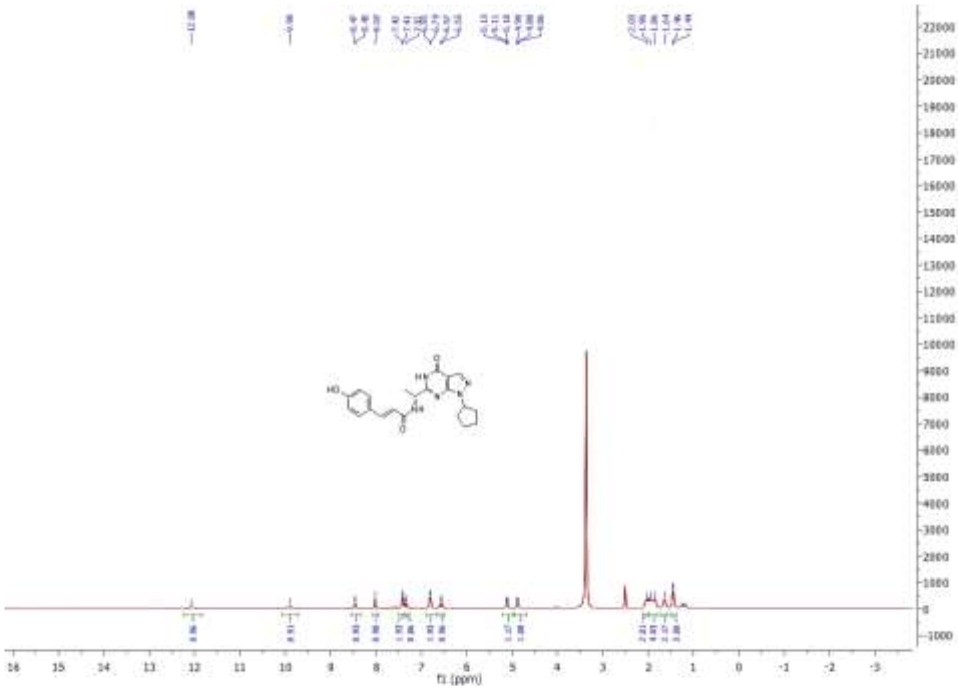

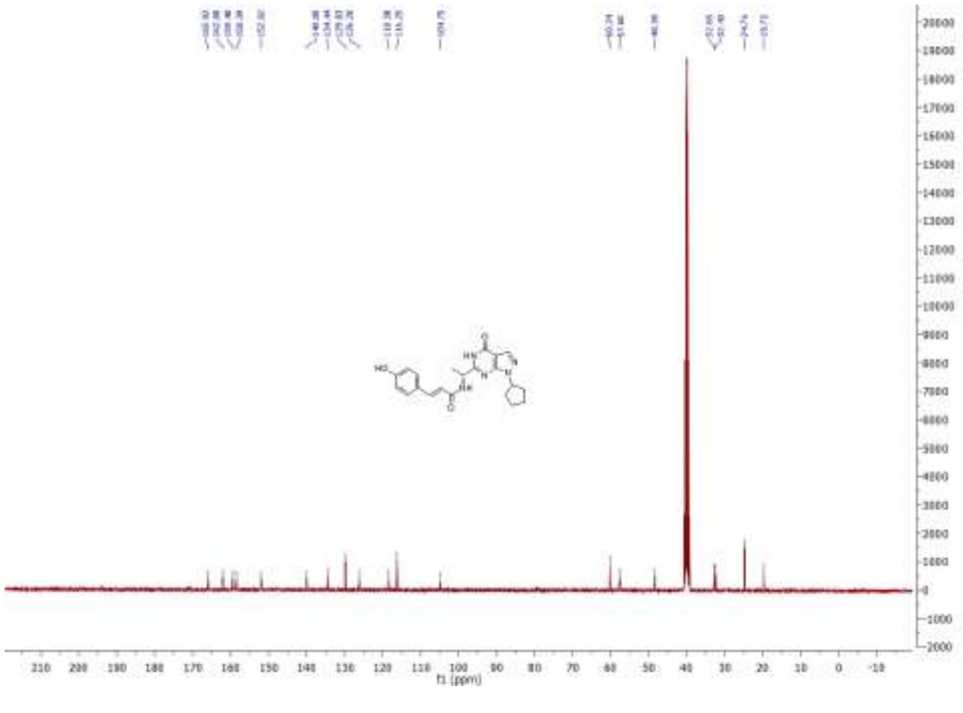

^{1}H and ^{13}C NMR of **1h**

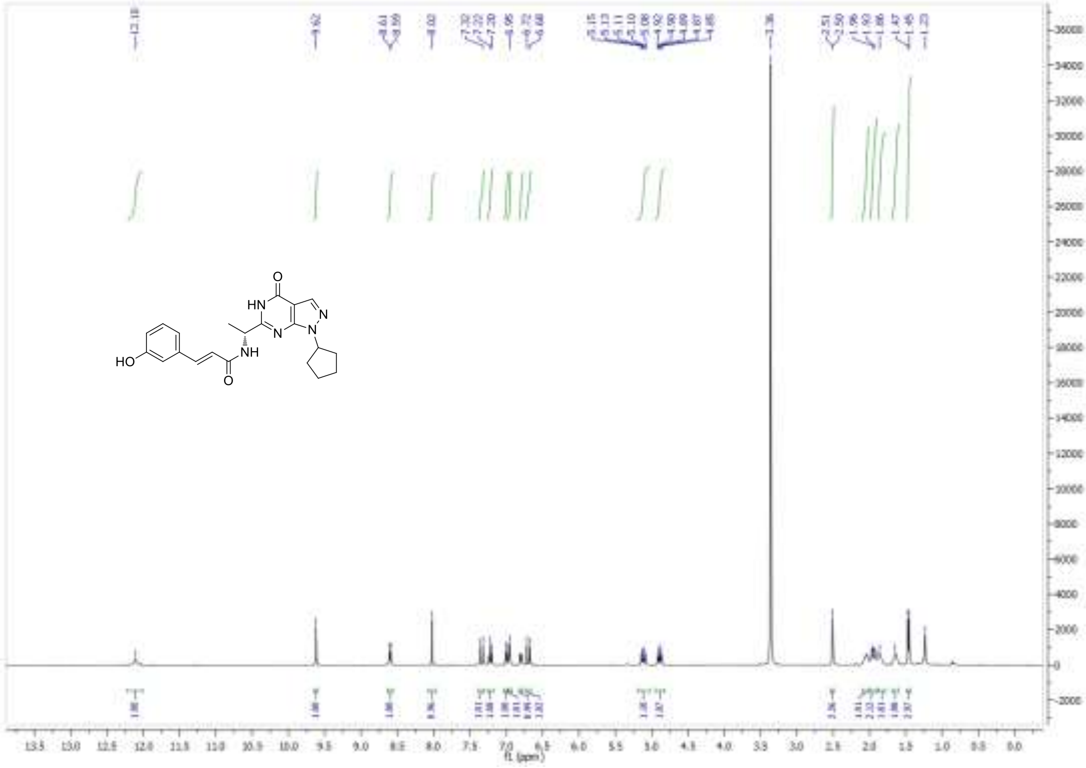

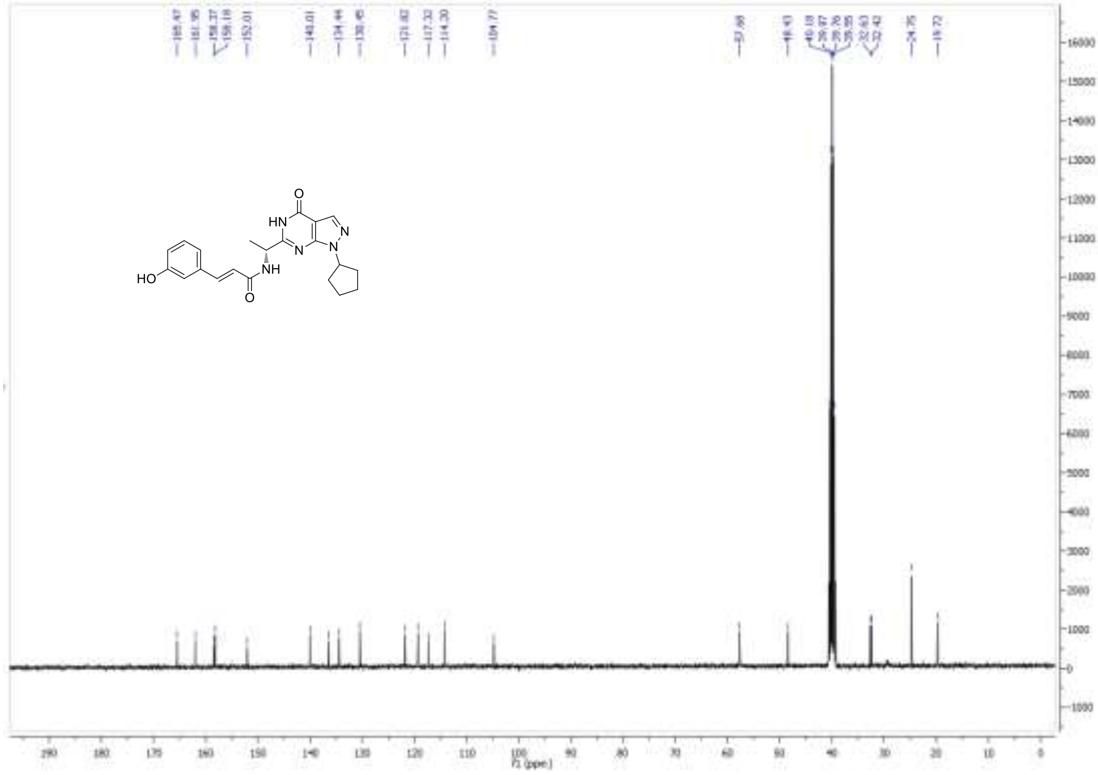

^{1}H and ^{13}C NMR of **1i**

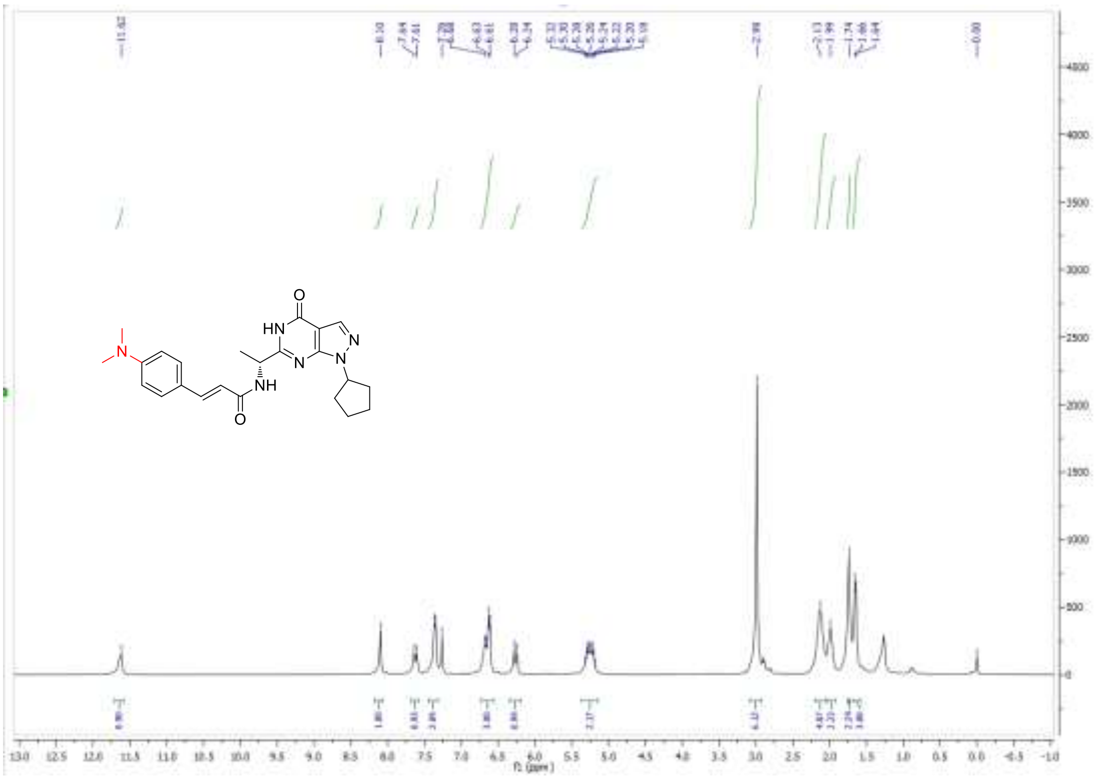

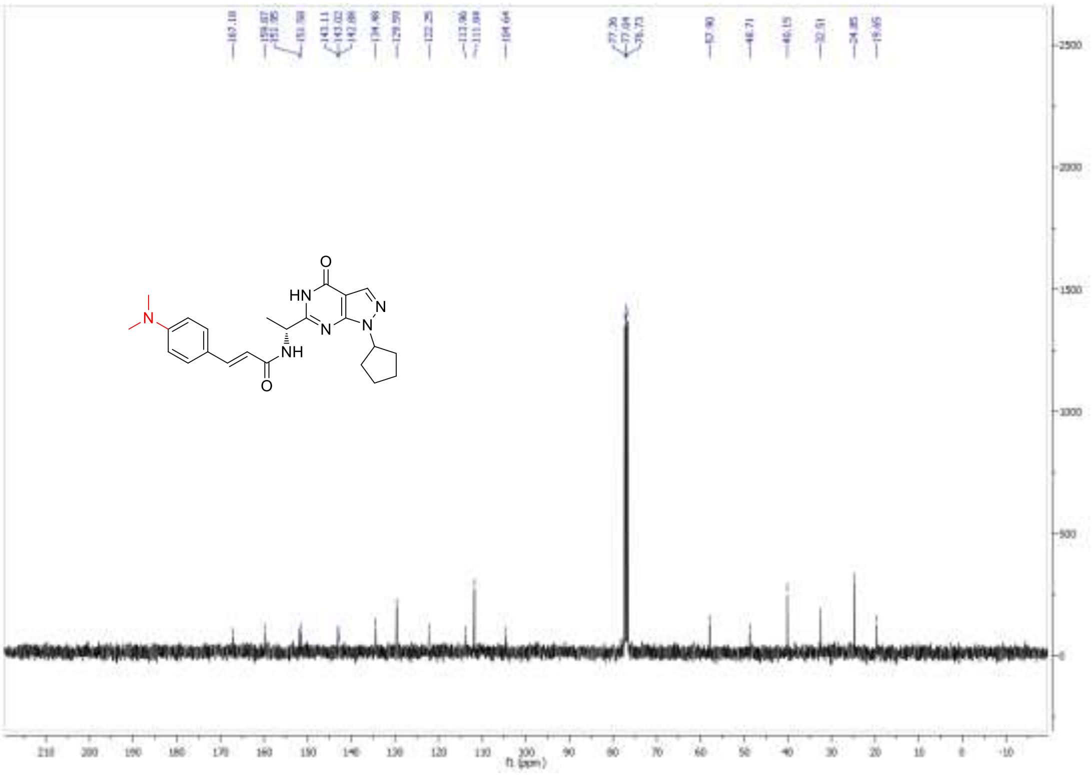

^{1}H and ^{13}C NMR of **1j**

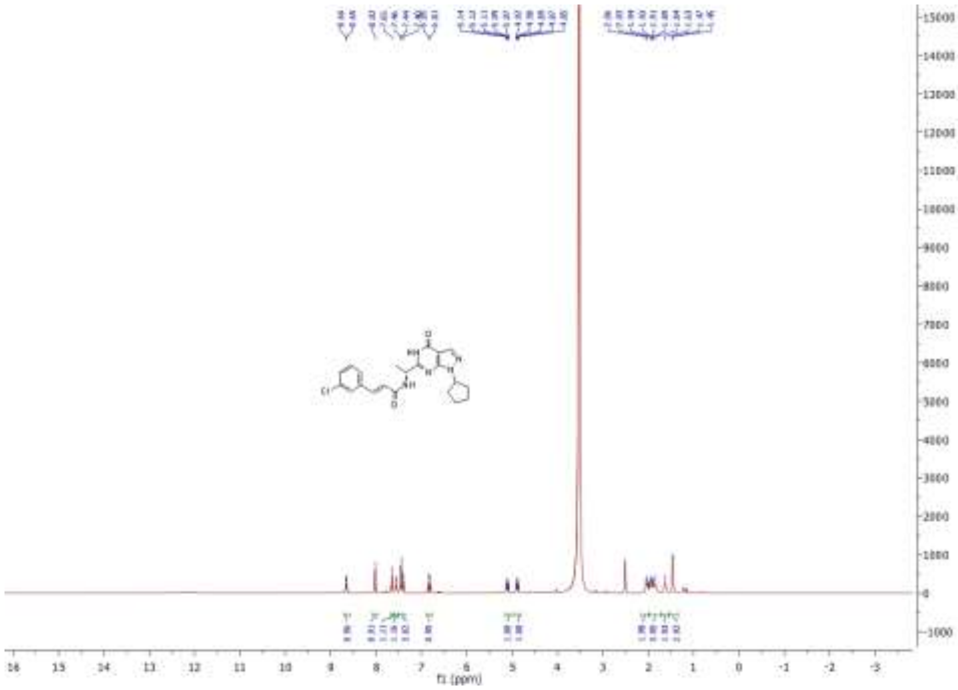

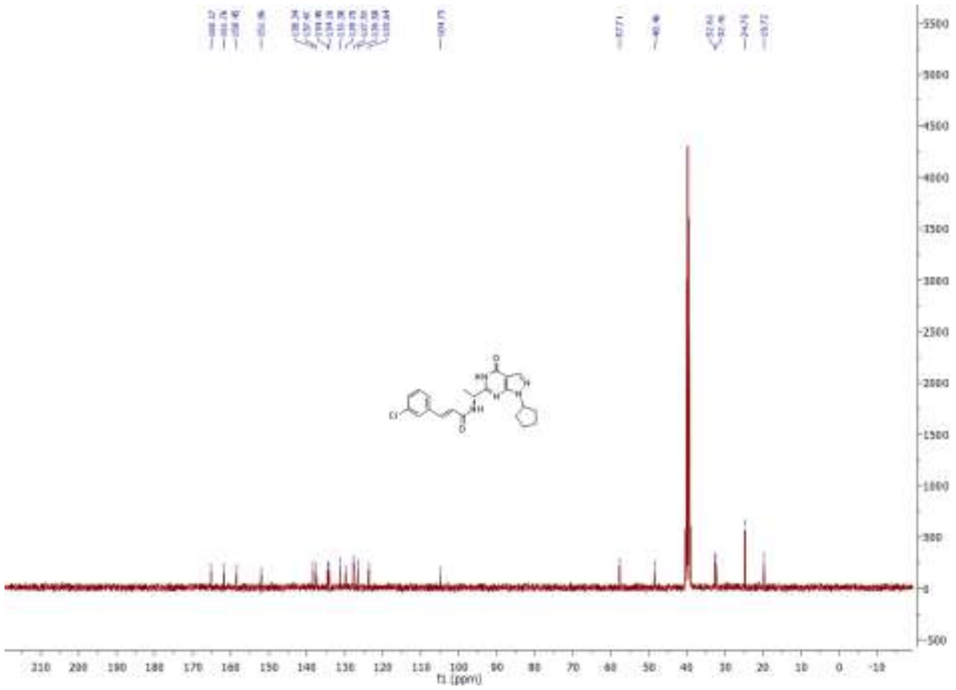

^{1}H and ^{13}C NMR of **1k**

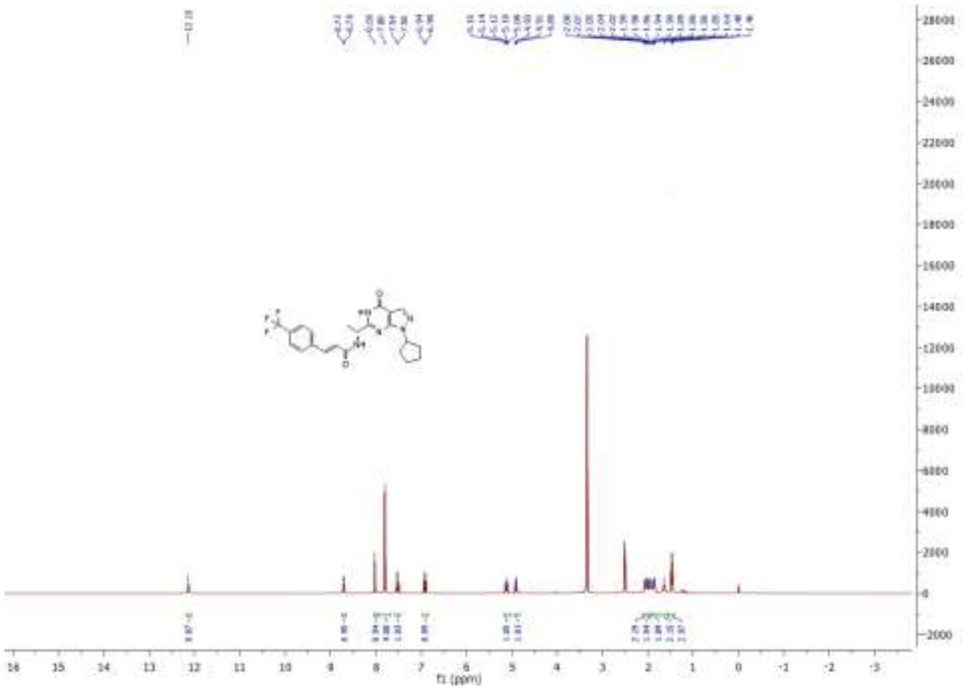

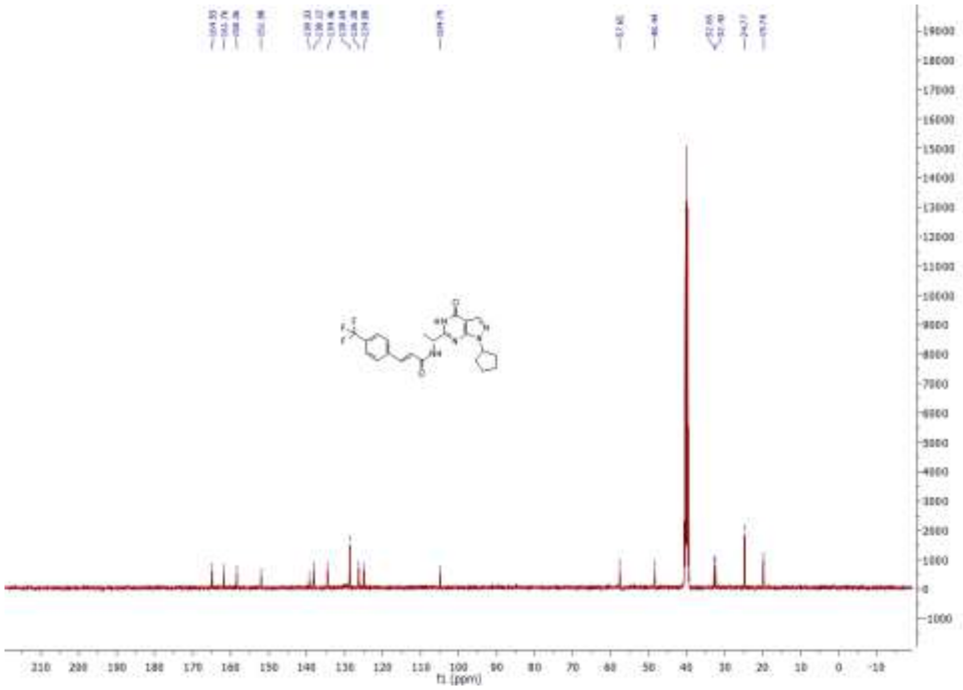

^{1}H and ^{13}C NMR of **1l**

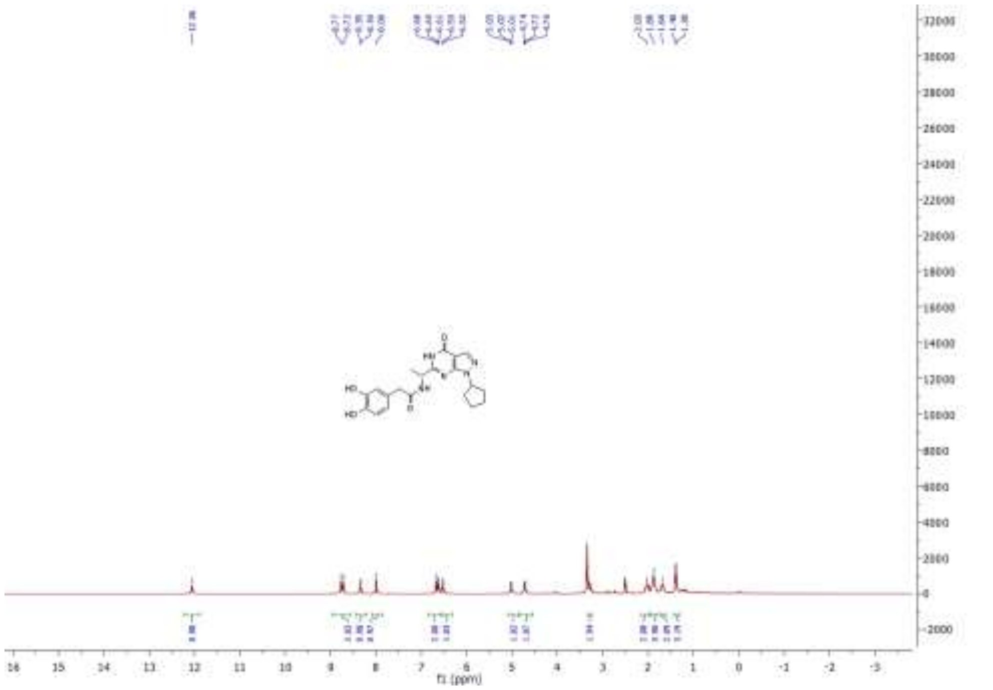

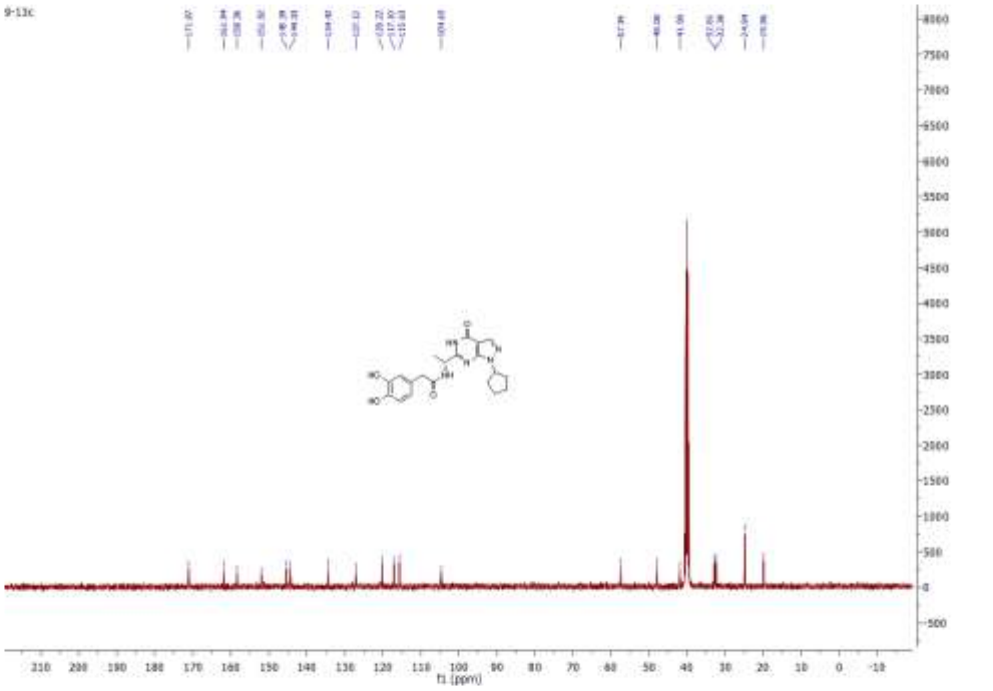

^1H and ^{13}C NMR of **2**

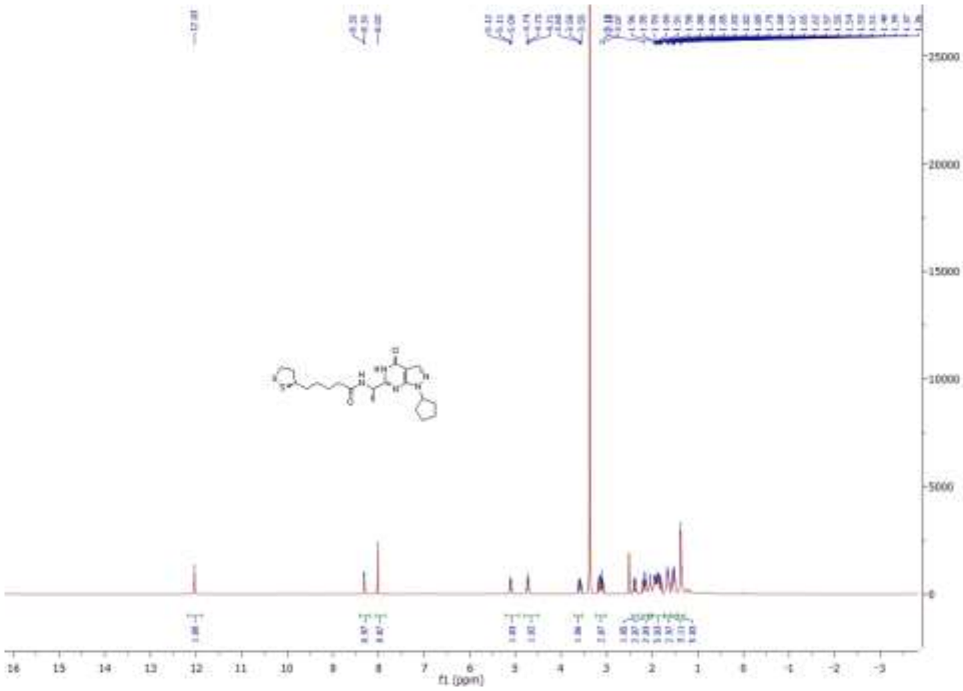

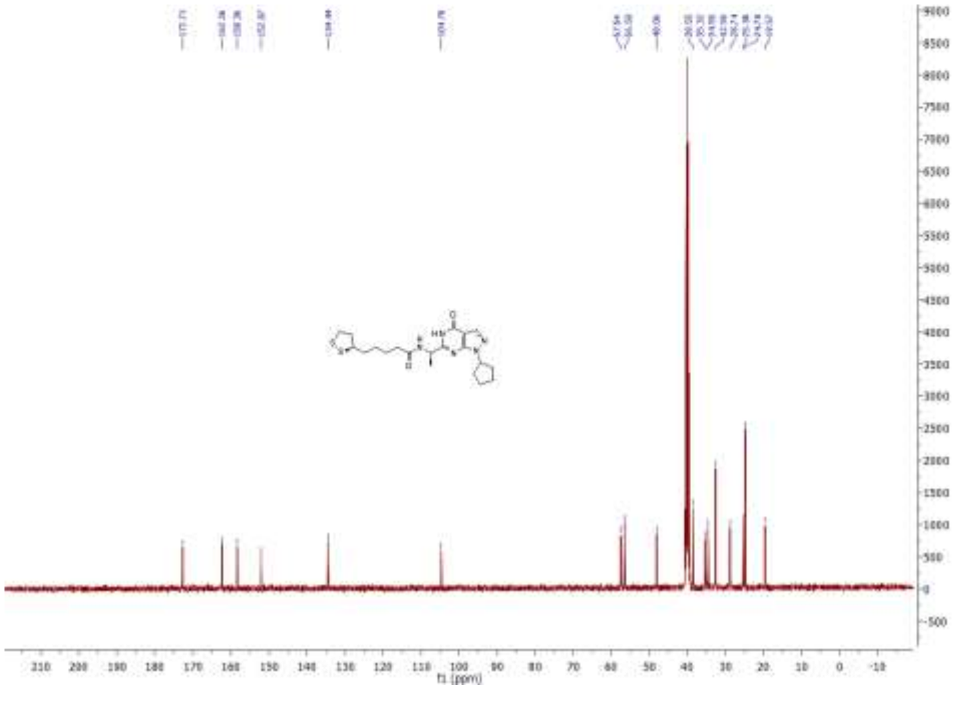

^{1}H and ^{13}C NMR of **3**

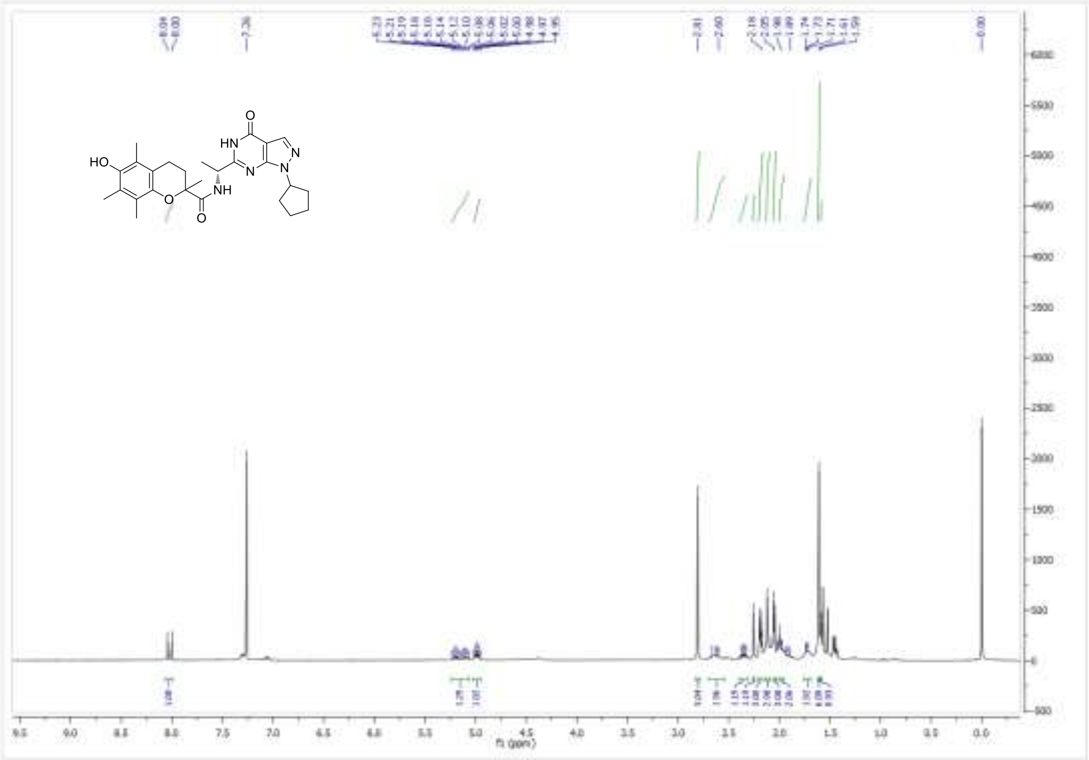

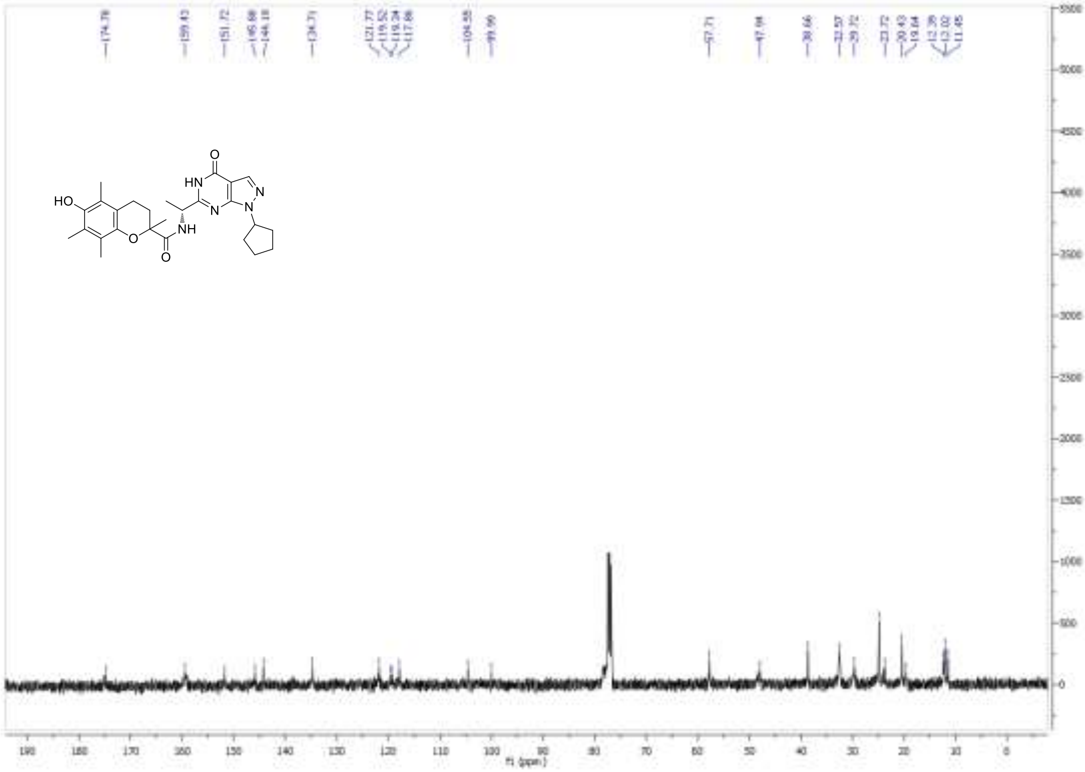

Supplement: IENZ_1412315_Supplementary_Material.pdf [file IENZ_A_1412315_SM2243.pdf]
